# Atkinesin-13A Modulates Cell-Wall Synthesis and Cell Expansion in *Arabidopsis thaliana* via the THESEUS1 Pathway

**DOI:** 10.1371/journal.pgen.1004627

**Published:** 2014-09-18

**Authors:** Ushio Fujikura, Lore Elsaesser, Holger Breuninger, Clara Sánchez-Rodríguez, Alexander Ivakov, Thomas Laux, Kim Findlay, Staffan Persson, Michael Lenhard

**Affiliations:** 1 Institut für Biochemie und Biologie, Universität Potsdam, Potsdam-Golm, Germany; 2 BIOSS Centre for Biological Signaling Studies, Faculty of Biology, Albert-Ludwigs-Universität Freiburg, Freiburg, Germany; 3 Max-Planck-Institut für Molekulare Pflanzenphysiologie, Potsdam-Golm, Germany; 4 Cell & Developmental Biology Department, John Innes Centre, Norwich Research Park, Norwich, United Kingdom; 5 ARC Centre of Excellence in Plant Cell Walls, School of Botany, University of Melbourne, Parkville, Victoria, Australia; University of Massachusetts at Amherst, United States of America

## Abstract

Growth of plant organs relies on cell proliferation and expansion. While an increasingly detailed picture about the control of cell proliferation is emerging, our knowledge about the control of cell expansion remains more limited. We demonstrate here that the internal-motor kinesin AtKINESIN-13A (AtKIN13A) limits cell expansion and cell size in *Arabidopsis thaliana*, with loss-of-function *atkin13a* mutants forming larger petals with larger cells. The homolog, AtKINESIN-13B, also affects cell expansion and double mutants display growth, gametophytic and early embryonic defects, indicating a redundant role of the two genes. *AtKIN13A* is known to depolymerize microtubules and influence Golgi motility and distribution. Consistent with this function, *AtKIN13A* interacts genetically with *ANGUSTIFOLIA*, encoding a regulator of Golgi dynamics. Reduced *AtKIN13A* activity alters cell wall structure as assessed by Fourier-transformed infrared-spectroscopy and triggers signalling via the *THESEUS1*-dependent cell-wall integrity pathway, which in turn promotes the excess cell expansion in the *atkin13a* mutant. Thus, our results indicate that the intracellular activity of AtKIN13A regulates cell expansion and wall architecture via THESEUS1, providing a compelling case of interplay between cell wall integrity sensing and expansion.

## Author Summary

Most of the visible growth of plant organs is driven by cell expansion without associated cell division. As plant cells are encased in cell walls, expansion requires the controlled loosening of the existing cell wall and synthesis of additional wall material. While a number of factors and plant hormones are known that promote cell expansion, what limits this process and thus restricts final cell and organ size is less well understood. Here, we identify a mutant that forms larger flowers because of increased cell expansion. The affected gene encodes a motor protein associated with the microtubule cytoskeleton that causes microtubule break-down and is required for ensuring an even distribution of secretory organelles within cells. Reduced activity of this motor protein triggers the activation of a pathway that detects defects in cell-wall integrity, which in turn leads to the observed increase in cell-wall synthesis and expansion. The *Arabidopsis* genome encodes another highly similar motor protein, and the combined loss of their activities causes severe defects, including reduced cell expansion. Thus, the two proteins fulfill an essential function in plant cell growth, and their full activity appears to be required to ensure normal cell-wall synthesis and a timely cessation of cell expansion.

## Introduction

Growth of plant lateral organs to their characteristic sizes is based on cell proliferation and on cell expansion [1]. In a first phase of organ growth cells throughout the primordium increase in size and divide mitotically. Cell proliferation then arrests progressively from the tip of the organ towards proximal regions, until all of the cells have ceased dividing and instead continue to grow by post-mitotic cell expansion. Genetic analysis in *Antirrhinum majus* and *Arabidopsis thaliana* has identified a number of factors that influence the final number of cells in an organ and thus its size [1]. By contrast, our knowledge about the factors regulating cell expansion in growing lateral organs is more limited [2]. Although it is well established that ploidy correlates with final cell size, the underlying molecular basis remains unclear [2], [3]. Whereas the phytohormones auxin, gibberellins and brassinosteroids can promote cell expansion, ethylene and jasmonic acid inhibit organ growth by affecting cell expansion [4], [5]. Brassinosteroids and gibberellins act via three antagonistic helix-loop-helix nuclear proteins to promote cell expansion [6], [7], and brassinosteroids also act via ARGOS-LIKE [8]. In addition, the TARGET OF RAPAMYCIN (TOR) signalling pathway in plants promotes cell expansion [9], [10], [11]. In petals, a specific isoform of the basic helix-loop-helix transcription factor BIGPETALp (BPEp) limits cell expansion and thus final petal size, acting downstream of jasmonic acid and in concert with the auxin response factor ARF8 [12], [13], [14].

Plant cells are encased by cell walls composed of cellulose, hemicelluloses and pectin that resist the turgor pressure of the cells and thus enable an erect growth habit [15]. For a cell to expand, its wall needs to be loosened in a controlled manner. Expansins are one class of cell wall-loosening factors. Increased or reduced expansin activity leads to larger or smaller organs due to enhanced or reduced cell expansion, respectively [16], [17], [18]. To prevent a progressive thinning of the wall during cell expansion, additional wall material needs to be synthesized and added to the growing wall. While hemicelluloses and pectins are synthesized in the Golgi apparatus, the cellulose microfibrils are produced at the plasma membrane by membrane-localized cellulose synthase (CESA) complexes [15], [19]. In higher plants these are thought to consist of up to 36 subunits drawn from a set of three different isoforms [19]. For example, the isoforms encoded by the *CesA1* (At4g32410), *CesA3* (At5g05170), and either *CesA2*, *CesA5* or *CesA6* (At5g64740) genes form the CESA complex for primary-cell wall synthesis in seedlings [20]. CESA complexes are presumably assembled in the Golgi in an inactive state and are transported to the plasma membrane where they become active [21], [22], [23], [24]. Delivery of CESA complexes to the plasma membrane can occur directly from the Golgi, or via small cytoplasmic compartments [21], [22], [25], to sites that preferentially co-occur with cortical microtubules. Once inserted in the plasma membrane, CESA complexes begin to polymerize cellulose microfibrils, which drives motility of the complexes along cortical microtubules, resulting in an ordered cellulose deposition on the inner face of the cell wall in a pattern that reflects the arrangement of the microtubules [23]. Movement of CESA complexes along microtubules requires POM-POM2/CELLULOSE SYNTHASE INTERACTING1, whose loss of function results in reduced cell expansion, similar to what is also observed for other mutants affecting cell-wall synthesis [26].

Two microtubule-based motors of the kinesin family appear to link the cortical array of microtubules to cell-wall synthesis [27], [28]. The FRAGILE FIBER1 (FRA1) protein, a member of the KIF4 family of kinesins, is required for the ordered deposition of cellulose microfibrils in the walls of interfascicular-fiber cells in the *Arabidopsis* stem [28]. Mutations in the internal-motor kinesin AtKINESIN-13A (AtKIN13A; At3g16630) lead to the outgrowth of an additional branch in *Arabidopsis* trichomes; this outgrowth of an extra branch most likely involves additional cell-wall synthesis [27]. The AtKIN13A protein is found in close association with Golgi stacks (and Golgi-derived secretory vesicles in peripheral root cap cells), and loss of its function causes clustering of Golgi stacks in the cell cortex [27], [29]. This suggests that AtKIN13A disperses Golgi stacks along cortical microtubules after they have been transported to the cell cortex via the actomyosin system [27], [30]. As in the *fra1* mutant, the distribution and arrangement of cortical microtubules are unchanged in *atkin13a* mutant leaf cells [27], [30]. Recent work has demonstrated that AtKIN13A has microtubule-depolymerizing activity *in vitro* and *in vivo*, and that this activity is required for a normal pattern of secondary cell-wall formation in xylem cells [31], [32]. Loss of *AtKIN13A* function results in smaller xylem cell-wall pits (i.e. regions free of secondary cell-wall deposition), while overexpression increases pit size, demonstrating that AtKIN13A influences cell-wall deposition in xylem cells. AtKIN13A protein is recruited to the plasma-membrane by interacting with MICROTUBULE DEPLETION DOMAIN1 (MIDD1) via its C-terminal coiled-coil domain; MIDD1 in turn is brought to the plasma membrane via active Rho of plant (ROP) GTPase signalling [31], [32]. However, how the activities of AtKIN13A in depolymerizing microtubules and dispersing Golgi stacks are connected is currently unknown. Based on the opposite trichome phenotypes of *atkin13a* and *angustifolia* (*an*) mutants [33], [34], it has been proposed that trichome branches are initiated at sites of preferential Golgi delivery [30]. *AN* (At1g01510) encodes a protein of the CtBP/BARS family that localizes to the *trans*-Golgi network [33], [34], [35]. Loss of *AN* function causes defects in Golgi-derived vesicles, suggesting that AN functions like BARS as a regulator of endomembrane trafficking.

The integrity of the cell wall during growth is actively monitored by plants [36]. One branch of the cell-wall integrity system comprises transmembrane receptor-like kinases (RLKs) that appear to sense changes in the structure and/or composition of the cell wall or the presence of fragments derived from cell-wall polymers. WALL-ASSOCIATED KINASES (WAKs) bind to pectin in the cell wall, and the WAK2 protein (At1g21270) has been suggested to link cell-wall sensing to the control of turgor pressure and ultimately cell expansion [37], [38], [39]. Three members of the *Catharanthus roseus* RLK1-LIKE (CrRLK1L) family from *Arabidopsis*, THESEUS (THE1; At5g54380), HERKULES-KINASE1 (HERK1) and FERONIA have also been implicated in cell-wall integrity sensing [40]. Under conditions of reduced cellulose biosynthesis in seedling hypocotyls, THE1 activity causes increased lignification and limits cell expansion by upregulating the expression of genes encoding cell-wall proteins, such as extensins, and pathogen-defense proteins [41]. By contrast, in wild-type plants THE1 activity is required redundantly with that of HERK1 to promote cell expansion, acting in parallel to the brassinosteroid pathway [42].

How cell expansion is limited to ensure an appropriate final cell and ultimately organ size remains an important open question. Also, the role of the cell wall in restricting final cell size is currently unclear. Therefore, we investigated a novel mutant that causes petal overgrowth due to excess cell expansion. Our results show that defects in cell-wall synthesis due to reduced *AtKIN13A* activity trigger *THE1*-dependent signalling, leading to changes in cell wall architecture and enhanced cell expansion. Thus, the structure of the cell wall appears to play an important role in limiting cell expansion during post-mitotic organ growth.

## Results

### Mutations in *AtKINESIN-13A* change petal size

**Figure 1:**
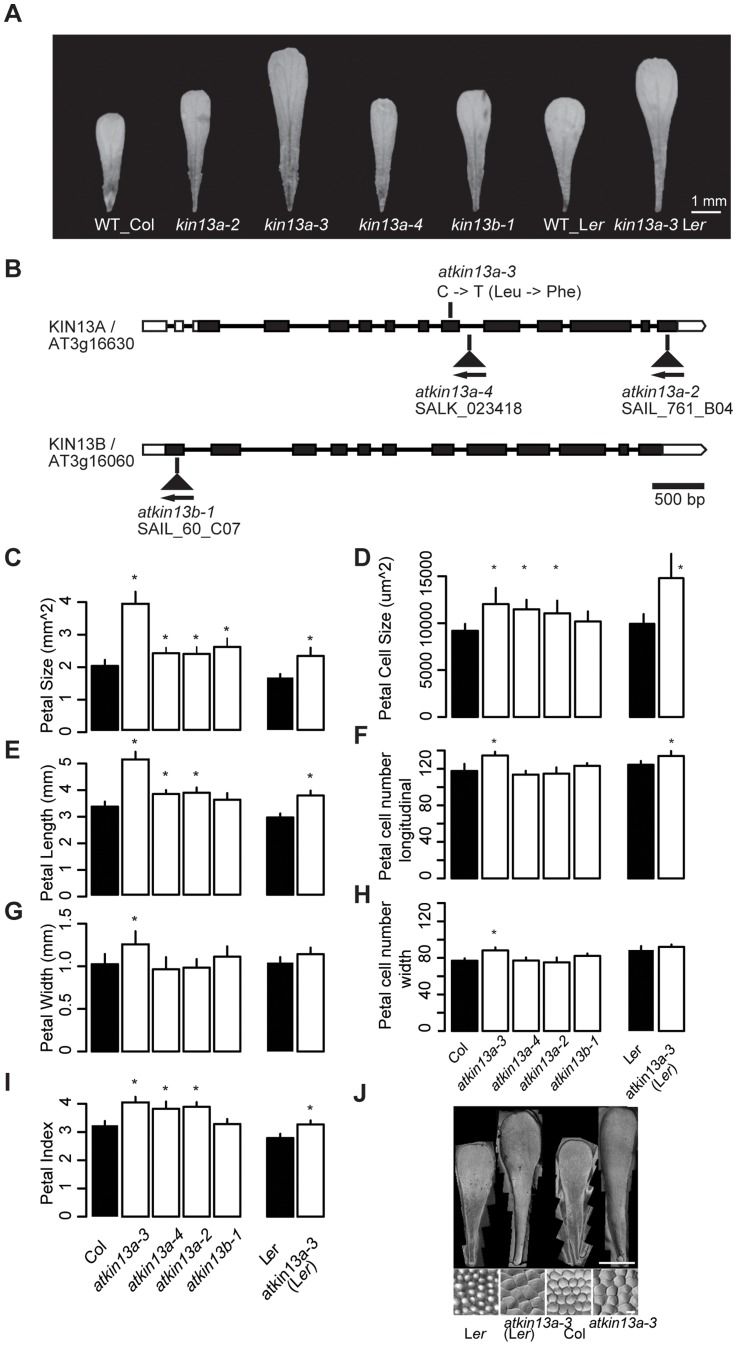
Petal phenotypes of *atkin13a* and *atkin13b* loss-of-function mutants. (A) Photographs of petals of the indicated genotypes. Scale bar is 1 mm. (B) Schematic representation of the *AtKIN13A* and *AtKIN13B* loci and position of mutant alleles. Open rectangles represent UTRs, filled rectangles show the protein-coding region, and thick connecting lines show introns. (C–I) Measurements of petal parameters for the indicated genotypes. Numbers in C and D indicate relative parameter values with respect to the corresponding wild-type values. Asterisk indicates significant difference from wild-type at p<0.05 (with Bonferroni correction for comparisons to Col-0). (C) Petal size. (D) Petal-cell size. (E) Petal length. (F) Petal cell number in the longitudinal direction. (G) Petal width. (H) Petal cell number along the petal width. (I) Petal index, i.e. length divided by width. (J) Gel prints (top) and representative cells (bottom) from petals of the indicated genotypes. Scale bars are 1 mm (top panel) and 100 µm (bottom panel). Values are mean + SD of 12 petals (C,E–I) or of 50 petal cells each from more than 10 petals (D).

In an EMS-mutagenesis screen in the Landsberg *erecta* (L*er*) background for mutations with altered petal size, we identified a line with larger petals (Fig. 1A). Genetic mapping and sequencing of candidate genes indicated that the mutation affects the *AtKINESIN-13A* (*AtKIN13A*; *At3g16630*) gene (Fig. 1B; Fig. S1). The mutant allele carries an EMS-induced C-to-T transition at position 1000 of the coding sequence, changing a leucine to a phenylalanine. The affected amino acid is part of an invariant Asp-Leu-Leu (DLL) motif in the central motor domain of KIN13 homologues from plant and animal kingdoms (Fig. S2H), suggesting that the mutation interferes with the known microtubule depolymerizing activity of the protein [31], [32]. Two mutant alleles of *AtKIN13A* carrying T-DNA insertions have previously been described as *atkin13a-1* (Salk_047048) and *atkin13a-2* (SAIL_761_B04) [27]; therefore, we named the EMS-induced allele *atkin13a-3*. In addition, we isolated another T-DNA insertion allele from the Salk collection [43] with the insertion in a more 5′ position than in *atkin13a-1* and will refer to this as *atkin13a-4* (line Salk_023418) (Fig. 1B). We used the *atkin13a-2*, *atkin13a-3*, and *atkin13a-4* alleles in this study. RT-PCR analysis indicated that *atkin13a-3* plants express full-length *AtKIN13A* mRNA, whereas no full-length transcript can be detected in mutants for the T-DNA insertion allele *atkin13a-4* (Fig. S2A).

We characterized the petal phenotype of the three *atkin13a* mutant alleles. To be able to better compare the effect of the *atkin13a-3* allele to that of the T-DNA insertion alleles in the Col-0 background, we introgressed it into the Col-0 background by four rounds of backcrossing; this line will be referred to as *atkin13a-3C*. The *atkin13a-3* allele leads to enlarged petals in both L*er* and Col-0 backgrounds (Fig. 1A,C). Length and width is similarly enlarged in the L*er* background, while in the Col-0 background petal length is more strongly affected, leading to an increased petal index (the ratio of length to width) as a description of petal shape (Fig. 1C,E,G,I). At the cellular level, cell numbers along the length and width directions of the petal are significantly, albeit weakly increased in *atkin13a-3* mutants, more strongly in the Col-0 introgression line (Fig. 1F,H), possibly reflecting the activity of accession-specific modifiers. More importantly, petal-cell size is significantly enlarged in the mutant, and in particular in the L*er* background the excess cell expansion accounts for essentially all of the overall increase in petal size (Fig. 1D,J). Measuring cell lengths along the petal in *atkin13a-3C* mutants indicates that cells are longer in the mutant than in wild type (Fig. S2B–D). The *atkin13a-3* allele behaves in a semi-dominant manner regarding petal size, with heterozygotes showing an intermediate increase in petal area relative to wild-type and homozygous mutant plants (Fig. S2E). In the T-DNA insertion mutants, petal size and petal length are also increased, albeit to a lesser extent (Fig. 1C,E). This results in an increased petal index, reflecting the narrower shape of the organs (Fig. 1A,E,I). Petal cell number is not significantly changed in the insertion mutants relative to wild type (Fig. 1F,H), yet petal cells in the lobe region are larger and longer (Fig. 1D, Fig. S2C,D).

Previous work has shown that *atkin13a-1* and *atkin13a-2* mutants form trichomes with more than the usual three branches [27]. Similarly, overbranched trichomes are observed in *atkin13a-3* and *atkin13a-4* mutants (Fig. S2F,G).

To determine whether the effects seen in the *atkin13a-3* and the *atkin13a-4* mutants are indeed the consequence of a loss of *AtKIN13A* function, we transformed a wild-type version of the gene into the homozygous mutants and tested for complementation of the phenotype. Trichome branching, petal size and petal-cell size were fully rescued in transformants in the *atkin13a-4* background (Fig. S3A–C). While transformants in the *atkin13a-3* background still formed significantly, albeit only slightly larger petals than wild type, their petal-cell size and trichome branching were fully rescued (Fig. S2A–C). The failure to completely rescue petal size likely reflects the semi-dominant nature of the *atkin13a-3* allele (see Discussion).

Thus, *AtKIN13A* function is required to limit cell expansion (and to a small degree also cell proliferation) in growing petals. The semi-dominant behaviour of the *atkin13a-3* allele suggests that the mutant protein that is most likely still produced from this allele acts as a dominant-negative form that interferes with the function of wild-type AtKIN13A and possibly other, closely related kinesins (see below).

### *AtKIN13A* function is required in late stages of petal growth to limit cell expansion

**Figure 2:**
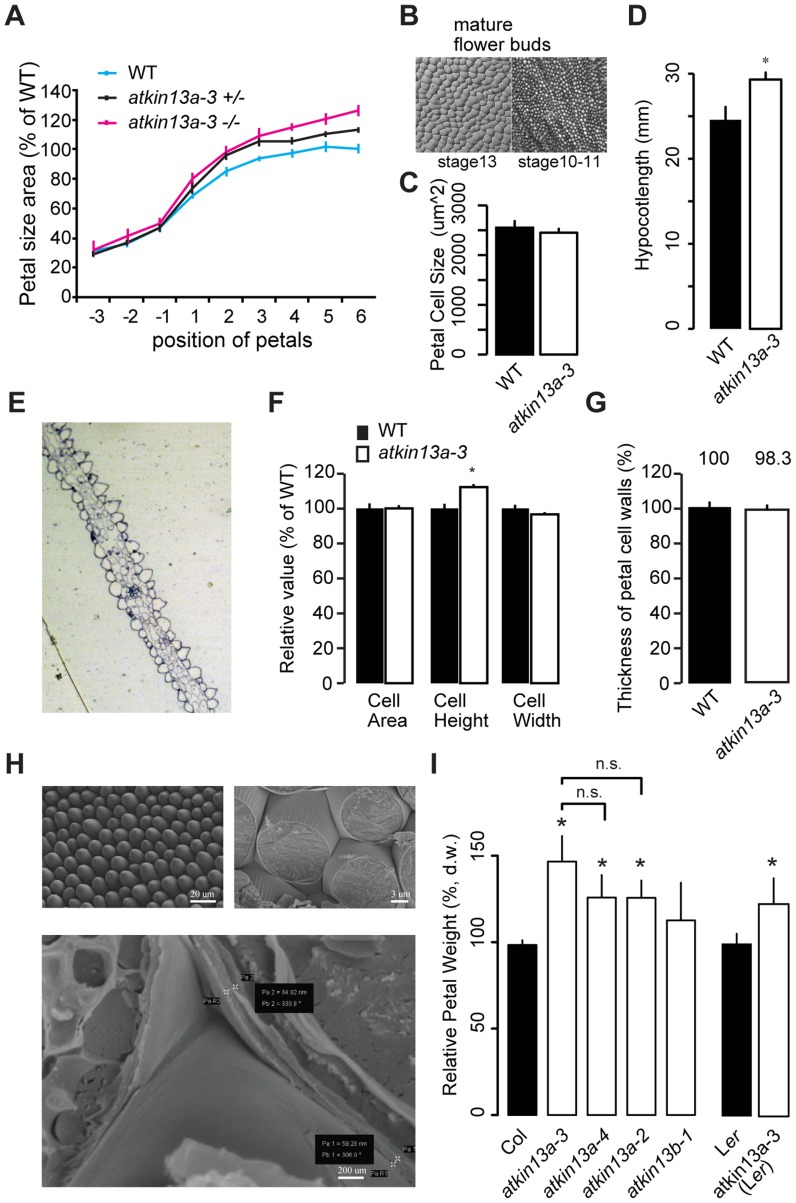
Increased postmitotic cell expansion in *atkin13a* mutants. (A) Developmental series of petal size for the indicated genotypes. Flower 1 represents the youngest open flower, while flower −1 is the oldest unopened flower bud. Values are normalized to the size of the wild-type petals from the oldest measured flowers. Values represent mean ± SEM (n<10 petals). (B) Gel-print images of wild-type petal cells from the indicated flower stages (after [71]). (C) Petal-cell size from stage 10–11 buds is not different between wild type and *atkin13a-3* mutants. Values are mean ± SD of 50 petal cells each from more than 6 petals. (D) Etiolated hypocotyls are longer in *atkin13a-3* mutants than in wild type. Values are mean ±SD of 10 plants. 8-day old seedlings were measured. (E) Toluidine-blue stained cross section through a mature wild-type (left) and a mature mutant petal (right). Adaxial side is to the right in both images. (F) Cross-sectional cell area, cell height and cell width from petals of wild type and *atkin13a-3^EMS^* mutants. Values are normalized to wild-type values and represent mean ± SD of 50 petal cells. (G) Thickness of petal-cell walls as measured from scanning-electron micrographs. Values are mean ± SEM of 100 petal cells from 10 petals, normalized to the wild-type value. (H) Scanning-electron micrographs of wild-type petals before (top left) and after (top right and bottom) freeze fracturing. Bottom image also indicates how measurements of cell-wall thickness were taken (pairs of white crosses). Length of scale bars is indicated. (I) Petal dry weight of the indicated genotypes. Values are mean + SD from 3 replicates of 50 petals each, normalized to the respective wild-type values. Differences between the three *atkin13a* mutant alleles are not statistically significant (n.s.). Asterisk indicates significant difference from wild-type at p<0.05 (with Bonferroni correction where appropriate).

The strong effect of the *atkin13a-3* mutation on petal-cell size prompted us to ask whether cell enlargement results from a decoupling of cell growth and cell division already in early stages of petal development, or whether the excess cell enlargement is only seen during the phase of postmitotic cell expansion. A comparison of petal-cell sizes from stage 10–11 petals shows no difference between *atkin13a-3* and wild-type petals (Fig. 2B,C). Similarly, following petal growth from the third-oldest unopened flower bud through to fully mature flowers indicates that petal size of the *atkin13a-3* mutant only deviates from wild type shortly before the flower opens up via strong expansion of petal cells (Fig. 2A). Thus, together these results suggest that *AtKIN13A* function is mainly required in the late phase of postmitotic cell expansion towards the end of petal growth to limit cell size.

The measurements of petal-cell sizes above were taken from gel-prints of petals and thus represent a measure of the two-dimensional ‘ground area’ of the cells [44]. To determine whether the cells in the *atkin13a-3* mutant are indeed larger in volume or whether it is merely their shape that has changed to a more flattened one despite a wild-type cell volume, we prepared transverse cross-sections through *atkin13a-3* mutant and wild-type petals and measured the average area, height and width of the conical cells in the petal lobe (Fig. 2E). The average height of *atkin13a-3* mutant petal cells was increased, while their width was slightly decreased compared to wild type; this results in the same cross-sectional area as in wild type (Fig. 2F), indicating that *atkin13a-3* mutant petal-cells are indeed larger in volume than wild-type cells. This result was further confirmed by measuring cell heights from confocal-microscopy images (Fig. S4C).

To determine whether at the cellular level the increased expansion is accompanied by more cell-wall synthesis or whether the same amount of cell-wall material is stretched out more thinly in the *atkin13a-3* mutant compared to wild type, we measured the thickness of the cell wall in cells of the petal lobe using two approaches. Firstly, we freeze-fractured petals to expose the lateral walls of the conical petal cells and measured the thickness of the walls using scanning electron microscopy (SEM) (Fig. 2G,H). Secondly, the transverse sections through the petal lobes (see above) were imaged using transmission electron microscopy, and the thickness of the cell walls at the base was measured. Both methods indicated that cell-wall thickness in the mutants was indistinguishable from that in the wild type, suggesting that mutant petal cells synthesize more cell-wall material that is stretched out to the same final thickness as in wild type (Fig. 2G; Fig. S4A,B). Increased synthesis of dry matter, much of which is cell-wall material [45], was also evident when comparing wild-type and *atkin13a* mutant petals (Fig. 2I). Dry weights correlated closely with overall petal area, with the strongest increase in *atkin13a-3* and lesser increases in the other two alleles (Fig. 1C, 2I).

To ask whether the increased cell expansion in *atkin13a-3* mutants is specific to petals, we determined the sizes of mesophyll cells in leaves. Mutants for *atkin13a* formed larger, yet fewer leaf cells (Supplemental Figure S3D), indicating that *AtKIN13A* function also limits cell expansion in leaves. In addition, *atkin13a-3* mutants formed longer hypocotyls in the dark (Fig. 2D). Hypocotyl elongation during etiolation relies exclusively on post-mitotic cell expansion [46], indicating that also in this situation *AtKIN13A* function is required to prevent excess cell expansion. Given the well-established relationship between cell size and ploidy levels [3], we asked whether the excess cell expansion in the mutant petals resulted from ectopic endoreduplication. However, flow cytometry on petal cells indicated no difference in ploidy levels (Fig. S4E).

Thus, *atkin13a-3* mutant petal cells deposit more cell-wall material, accompanied by excess cell expansion to a larger final volume without any increase in ploidy levels.

### *AtKIN13A* interacts genetically with *AN*

To place *AtKIN13A* in genetic-interaction networks, we generated double mutants between *atkin13a* and other mutants showing a defect in cell expansion and/or petal growth [47], [48], [49]. Double mutants of *atkin13a-3* and *ethylene insensitive2* (*ein2*), *brassinosteroid insensitive1* (*bri1*) or *rotundifolia3* (*rot3*; a mutant with reduced, but not abolished levels of active brassinosteroids; [50]), showed essentially additive phenotypes regarding their petal sizes (Fig. S5A–C), suggesting that *AtKIN13A* acts independently of ethylene and brassinosteroids.

**Figure 3:**
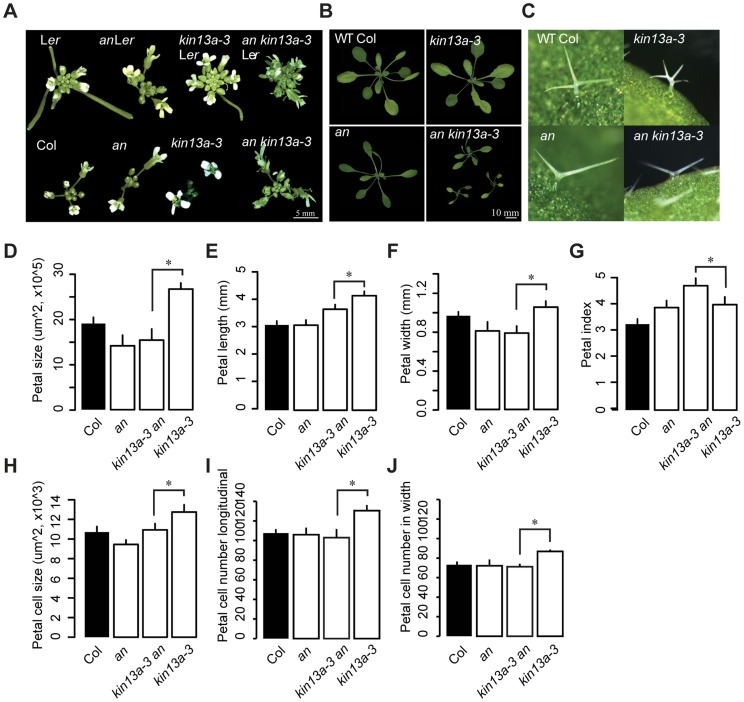
Genetic interaction between *an* and *atkin13A*. (A) Photographs of inflorescences of the indicated genotypes. As the *atkin13a-3* mutant is from the L*er* background and *an-1* from the Col-0 background, double and single mutants were selected either with (top row) or without functional *ERECTA* (bottom row). Scale bar is 5 mm. (B) Photographs of rosettes Scale bar is 10 mm. (C) Photographs of leaf trichomes of the indicated genotypes. (D–J) Measurements of petal parameters for the indicated genotypes. (D) Petal size. (E) Petal length. (F) Petal width. (G) Petal index, i.e. length divided by width. (H) Petal-cell size. (I) Petal cell number in the longitudinal direction. (J) Petal cell number along the petal width. Values are mean ± SD of 12 petals from more than 8 plants (A–F,H,I) or of 50 petal cells from more than 8 petals (G). Asterisk indicates significant difference at p<0.05.

The *an atkin13a-3* double mutant showed a novel phenotype not observed in either single mutant. Double mutant rosettes are smaller than in either single mutant (Fig. 3B). The inflorescences have a tousled appearance, with an unordered arrangement of floral organs (Fig. 3A). Petals of the double mutant had the same area as *an* single mutant petals, yet their shape was longer and narrower than in either single mutant (Fig. 3D–G). While the increase in petal-cell numbers observed in *atkin13a-3* mutants is suppressed by introducing the *an* mutation, the size of double mutant petal cells was intermediate between that of the single mutants (Fig. 3H–J). Regarding trichome branching, the *an* mutation is epistatic over *atkin13a-3*, with double mutants forming only two-branched trichomes (Fig. 3C). Thus, *an* and *atkin13a-3* show a non-additive interaction in several respects, with the double mutant presenting a synergistic (e.g. rosette growth, inflorescence organisation) or epistatic phenotype (e.g. trichome branching) in different processes. This suggests that at least in these processes *AN* and *AtKIN13A* interact to control cell growth via their effects on Golgi function.

### The THESEUS-dependent cell-wall integrity pathway is activated in *atkin13a* mutants, resulting in increased cell expansion

**Figure 4:**
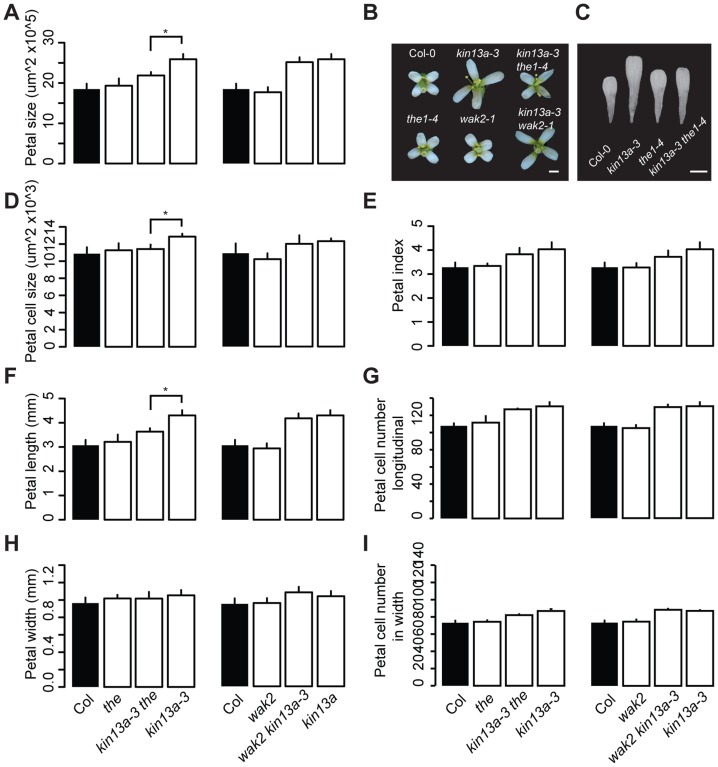
Excess cell expansion in *atkin13a* mutants requires *THE1* activity. (A,D–I) Measurements of petal parameters for the indicated genotypes. (A) Petal size. (B) Whole-flower photographs of the indicated genotypes. (C) Photographs of petals of the indicated genotypes. Scale bars are 1 mm in (B,C). (D) Petal-cell size. (E) Petal index, i.e. length divided by width. (F) Petal length. (G) Petal cell number in the longitudinal direction. (H) Petal width. (I) Petal cell number along the petal width. Values are mean ± SD of more than 12 petals from 8 plants (A,E–I) or of 50 petal cells from more than 8 petals (D). Asterisk indicates significant difference at p<0.05.

The above results indicate that the increased cell expansion in *atkin13a* mutant petals is accompanied by increased synthesis of cell-wall material. We wondered how a defect in this kinesin could cause increased cell-wall deposition, especially in light of its known role in microtubule depolymerization and ensuring a uniform distribution of Golgi stacks in the cells [27], [29]. One possibility is that a spatially less uniform cell-wall deposition in *atkin13a* mutants is perceived by cell-wall integrity sensing, and that this secondarily causes an upregulation of cell-wall synthesis and increased cell expansion. To test this idea, double mutants were analyzed between *atkin13a-3C* and *the1-4* or *wak2-1* mutants [38], [41], [42]. Based on published work, both the *wak2-1* and the *the1-4* alleles represent likely null-mutant alleles, from which no full-length mRNA can be detected [38], [42], making them suitable for epistasis analysis. When grown on soil, neither the *wak2-1* nor the *the1-4* single mutants have any morphological phenotypes at the whole-plant [38], [42] or the flower level (Fig. 4). The *atkin13a-3C wak2* double mutant was very similar to *atkin13a-3C* single mutants with respect to petal area, cell number and cell size, suggesting that petal overgrowth in *atkin13a-3C* does not require *WAK2* activity (Fig. 4A,B,D–I). By contrast, petal size of the *atkin13a-3C the1-4* double mutant was significantly smaller than that of the *atkin13a-3C* single mutant (Fig. 4A–C). At the cellular level, cell numbers in double mutant petals along the length and width directions are similar to the *atkin13a-3C* single mutant (Fig. 4F–I); by contrast, the increased cell expansion is entirely suppressed in the double mutant, with cell sizes indistinguishable between *the1-4* single and the double mutant (Fig. 4D). This indicates that *THE1* activity is required for the increased cell size in *atkin13a* mutants, suggesting that activation of the *THE1*-dependent cell-wall integrity pathway triggers the increased cell-wall synthesis and cell expansion as a secondary consequence of an unknown initial cell wall-related defect due to the *atkin13a* mutation.

### Cell-wall structure is changed in *atkin13a* mutants as a result of *THE1* activation

**Figure 5:**
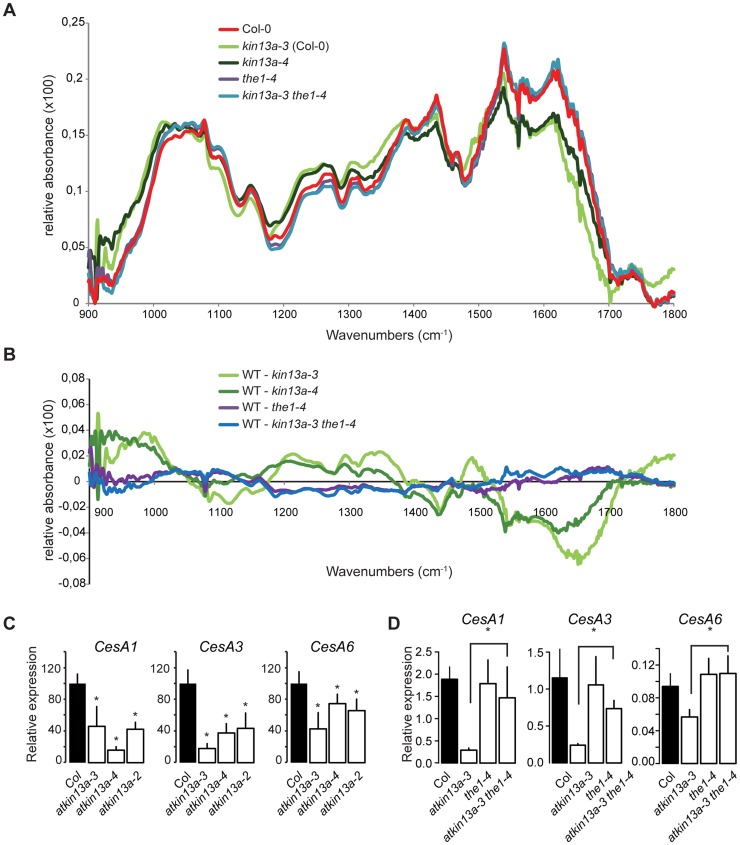
Cell-wall composition is altered in *atkin13a* mutants. (A) Average Fourier-Transformed-Infrared (FTIR) spectra of three independent biological replicates of the indicated genotypes. (B) Difference spectra obtained by digital subtraction of the average spectra of the indicated genotypes from the Col-0 average spectrum. (C,D) Expression of genes encoding CesA subunits required for primary-cell wall formation in the indicated *atkin13a* mutant alleles (C) and in single and double mutants of *atkin13a-3* and *the1-4* (D). Values are mean + SD of three biological replicates. Asterisk indicates significant difference from wild-type at p<0.05 (with Bonferroni correction).

To test whether in addition to the amount of cell-wall material its composition was also changed in *atkin13a* mutants, we analyzed cell-wall structure and composition by Fourier-transformed infrared-spectroscopy (FTIR) and biochemical profiling of cell-wall monosaccharides. As the latter requires a larger amount of dried wall material than can feasibly be obtained from petals, we performed these analyses using leaf material. As shown above, leaf cells are similarly enlarged in *atkin13a* mutants, allowing us to use leaves as meaningful proxies for petals for the cell-wall assays. We analyzed two independent *atkin13a* mutant alleles in the Col-0 background (*atkin13a-3C* and *atkin13a-4*), *the1-4* single and *atkin13a-3C the1-4* double mutants. FTIR was performed on purified cell-wall preparations. Both independent *atkin13a* mutant alleles showed a very similar difference compared to the wild type, with higher signals in the range from 920 to 1050 cm^−1^ and much lower signals in the range from 1550 to 1700 cm^−1^ (Fig. 5A,B, Fig. S6A,B). Comparing the *the1-4* single and *atkin13a-3C the1-4* double mutants revealed an almost indistinguishable FTIR profile that was very similar to that of the Col-0 wild type, demonstrating complete epistasis of the *the1-4* mutation. Biochemical profiling of monosaccharides after cell-wall hydrolysis did not show significant differences between the five tested genotypes, except for higher non-cellulosic glucose levels in *atkin13a-3C* single and *atkin13a-3C the1-4* double mutants (Table 1). To determine whether the organization of the cell wall was affected, petals from unopened flowers (stage 10–11) were stained with Pontamine Fast Scarlet 4B [51] as described in [52] to visualize cellulose microfibrils (Fig. S7). In most of the petal cells we were unable to discern any clear microfibrils, possibly due to background signals from hemicelluloses and/or the nature of petal-cell walls. No clear differences in the staining patterns between wild-type and mutant petals were detectable (Fig. S7).

**Table 1:** Monosaccharide composition of cell walls from 20-days old leaves.

	Glucose		Galactose		Manose		Xylose		Arabinose		Fucose		Rhamnose		Cellulose (Glc)		UronicAcids	
	Av	SE	Av	SE	Av	SE	Av	SE	Av	SE	Av	SE	Av	SE	Av	SE	Av	SE
Col-0	7,804	0,465	21,398	2,296	5,868	0,146	17,996	0,908	12,226	0,319	6,177	2,449	13,068	1,405	131,503	9,925	74,486	5,231
*kin13a-3*	**9,998**	0,429	23,023	0,827	6,826	0,613	20,138	0,513	13,179	1,236	6,832	1,772	15,202	0,322	145,307	1,702	69,039	3,929
*kin13a-4*	9,315	1,457	19,770	2,072	5,809	1,004	17,216	1,858	12,089	1,540	3,985	0,526	13,192	2,053	118,046	8,765	64,288	1,143
*the1-4*	10,168	0,531	21,527	0,486	6,355	0,203	20,565	0,215	11,725	0,466	3,936	0,382	15,089	0,467	144,538	2,965	68,538	1,981
*kin13a-3 the1-4*	**9,400**	0,200	22,144	1,064	5,601	0,077	18,892	0,600	11,196	0,426	3,821	0,195	13,990	0,068	123,293	3,792	73,028	2,724

Alcohol-insoluble residues (AIR) were prepared from 20-days old leaves of the indicated genotypes. The results are given as average (µg/mg of AIR) of three independent biological replicates (±SE). Numbers in bold indicate significant differences between the respective mutant and Col-0 wild type (Student's t-test, p<0.05).

Supporting an effect of the *atkin13a* mutations on cell-wall structure, the expression of the *CesA1*, *CesA3*, and *CesA6* genes encoding primary cell wall CesA subunits was strongly reduced in *atkin13a* mutant inflorescences compared to wild type (Fig. 5C). To determine whether also these gene-expression phenotypes of *atkin13a* mutants are dependent on activation of the *THE1*-dependent pathway, we assessed expression of the *CesA* genes in *the1-4* single and *atkin13a-3 the1-4* double mutants. Expression of all three genes was very similar in *the1-4* single mutants compared to wild type (Fig. 5D). Also, expression of the CesA subunit genes is not significantly different between *the1-4* single and *the1-4 atkin13a-3* double mutants (Fig. 5D), but is significantly higher in the double mutants than in the *atkin13a-3* single mutant. This suggests that the reduction of *CesA* gene expression in *atkin13a* mutants results to a large extent, if not entirely, from activation of the *THE1* pathway.

By contrast, the *the1-4* mutation does not rescue the trichome-overbranching phenotype of the *atkin13a* mutant, despite detectable expression of *THE1* in trichomes based on publicly available microarray data (Fig. S6C; http://bar.utoronto.ca/efp/cgi-bin/efpWeb.cgi; [53]).

Thus, the structure of the cell wall, though not its overall biochemical composition, appears to be affected by alterations in *AtKIN13A* activity. Reduced activity triggers signalling via the *THE1* pathway, resulting in the modified cell-wall structure and increased cell expansion.

### *AtKIN13A* and its close homologue *AtKIN13B* are redundantly required to promote cell expansion

The *Arabidopsis thaliana* genome contains a closely related gene to *AtKIN13A*, termed *AtKIN13B* (*At3g16060*). This locus codes for a protein with 62% identity/74% similarity to AtKIN13A in the internal motor domain and 58% identity/82% similarity to AtKIN13A in a region of 84 amino acids at the very C-terminal end of the proteins. Based on publicly available gene expression information, both genes show a very similar gene expression pattern, including expression in trichomes (http://bar.utoronto.ca/efp/cgi-bin/efpWeb.cgi; [53]). To determine whether *AtKIN13A* and *AtKIN13B* fulfill similar functions in plant growth, we isolated a T-DNA insertion in *AtKIN13B*, termed *atkin13b-1* (SAIL_60_C07) (Fig. 1B). No full-length transcript is detectable in homozygous mutants (Fig. S2A). Plants with the *atkin13b-1* mutation also form significantly larger petals, with cell size tending to be larger than wild type, even though this difference is not statistically significant; however, in contrast to *atkin13a* mutants, petal shape of *atkin13b-1* mutants is not affected, as indicated by the unchanged petal index (Fig. 1A,C,I). The size of leaf-mesophyll cells was also increased in *atkin13b-1* mutants (Fig. S4D). However, in contrast to *atkin13a* mutants, these plants do not form overbranched trichomes (Fig. S2F).

**Figure 6:**
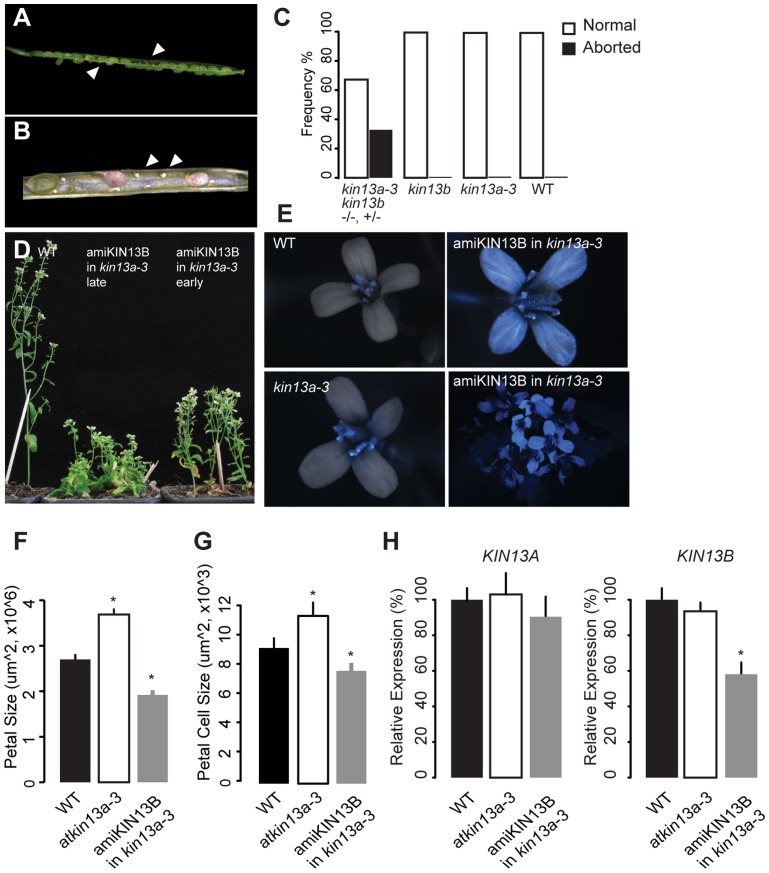
Redundant function of *AtKIN13A* and *AtKIN13B*. (A,B) Lower-magnification (A) and higher-magnification view of opened siliques of an *atkin13a-3 +/atkin13a-3 atkin13b-1* plant to show seed abortion (arrowheads in (A)) and unfertilized ovules (arrows in (B)). (C) Quantification of seed abortion. Values are based on more than 100 seeds per genotype. (D) Whole-plant photographs of the indicated genotypes after EtOH-induction before (early) and during bolting (late). (E) Overlays of light and CFP fluorescence micrographs of flowers from the indicated genotypes after EtOH-induction. CFP fluorescence indicates expression of the amiRNA transgene. The two images on the right show a single flower and an overview of an inflorescence from an *amiKIN13B*-expressing *atkin13a-3* mutant plant. (F) Petal sizes of indicated genotypes. Values are mean ±SD of 12 petals from more than 8 plants. (G) Petal-cell sizes of the indicted genotypes. Values are mean ± SD of 50 petal cells from more than 16 petals. (H) Relative expression of *AtKIN13A* (left) and *AtKIN13B* (right) in plants of the indicated genotypes after EtOH induction. Values are mean ± SD of three technical replicates. A biological replicate experiment is shown in Fig. S8D. Asterisk indicates significant difference from wild-type at p<0.05 (with Bonferroni correction).

*AtKIN13A* and *AtKIN13B* code for similar proteins, raising the possibility that they might act redundantly. To test this, we sought to generate plants lacking the activity of both genes. The two loci are tightly linked on chromosome 3, separated by approximately 210 kb. Therefore, in the progeny of *atkin13a-3 +/+ atkin13b-1* transheterozygous plants, we searched for recombinant chromosomes carrying the mutant alleles at both loci by screening for plants that are homozygous mutant for one and heterozygous for the other locus. Out of more than 1000 progeny plants, one *atkin13a-3 +/atkin13a-3 atkin13b-1* plant was identified. Amongst its progeny, however, no doubly homozygous mutant could be identified; instead, in the siliques of *atkin13a-3 +/atkin13a-3 atkin13b-1* plants many ovules were not fertilized, and of the developing seeds over 25% were brown and shrivelled, indicative of both a gametophytic defect and embryo lethality (Fig. 6A,B). Thus, *AtKIN13A* and *AtKIN13B* indeed seem to act redundantly and to fulfil an essential function for gametophyte and early plant development.

As an alternative approach to characterizing the effects of combined *AtKIN13* loss of function, a construct for AlcR/*AlcA*-mediated EtOH-inducible expression of an artificial microRNA (amiRNA) against *AtKIN13B* was introduced into the *atkin13a-3* background, and an analogous construct expressing an amiRNA against *AtKIN13A* was introduced into *atkin13b-1* mutants [54], [55]. For both constructs, EtOH-induction can be monitored with a linked *AlcA::CFP* reporter gene on the same T-DNA (*35S::AlcR—AlcA::miRNA—AlcA::CFP*; Fig. 6E, Fig. S8A). Inducing expression of the amiRNA before and during bolting results in reduced stem elongation (Fig. 6D). Similarly, amiRNA expression during flower development leads to the formation of smaller petals due to reduced cell expansion (Fig. 6F,G, Fig. S8B). Successful downregulation of the target mRNA was confirmed by qRT-PCR (Fig. 6H).

Thus, together these observations indicate that the two related AtKIN13 proteins are essential for normal cell expansion. They also suggest that while a moderate reduction in *AtKIN13* function triggers excess cell enlargement, a stronger reduction causes more severe defects in cell development, resulting in reduced cell expansion and ultimately gametophyte and embryo lethality.

## Discussion

### *AtKIN13* activity is essential for plant growth

*AtKIN13A* and *AtKIN13B* encode internal-motor kinesins that associate with microtubules [27], [56]. AtKIN13A has been demonstrated to depolymerize microtubules both *in vitro* and *in vivo*, and this activity depends on its motor domain [31], [32]. Consistent with this, the strong mutant phenotype seen in the *atkin13a-3* allele results from an amino-acid exchange in a highly conserved motif in the central motor domain (Fig. 1; Fig. S1). This mutation behaves in a semi-dominant manner and causes a more severe phenotype than what is observed in available T-DNA insertion lines. Animal kinesin-13 proteins act as dimers to depolymerize microtubules, with dimerization mediated by their N-terminal and coiled-coil domains [57], [58]; thus, a plausible explanation for the stronger phenotype than in the T-DNA insertion lines is that the mutated protein behaves in a dominant-negative manner by binding to wild-type AtKIN13A (in heterozygous plants) or AtKIN13B protein molecules (in *atkin13a-3* homozygous mutants) and inactivating the resulting dimers, or by blocking access of AtKIN13B dimers to microtubule ends in *atkin13a-3* homozygous mutants. This notion assumes that the two proteins share the same microtubule depolymerization activity, and are targeted to the same subcellular regions, such that in an *atkin13a* knock-out mutant the AtKIN13B protein takes over a large part of its function, leading to only a subtle phenotype. While not yet demonstrated experimentally, we believe this is a plausible assumption, as both protein domains with a known function (the internal motor domain and the C-terminal region that encompasses the coiled-coil domain and the region binding to MIDD1; [56], [58]) are very similar between AtKIN13A and AtKIN13B proteins, suggesting they have the same molecular function, can heterodimerize and both bind to MIDD1. Indeed, functional redundancy between the two genes is evident from the much stronger phenotypes seen in double mutant situations compared to either single mutants (Fig. 6). Embryos carrying homozygous loss-of-function mutations at both loci were not viable, indicating that *AtKIN13* function is essential for embryo development. Similarly, downregulating *AtKIN13A* expression in an *atkin13b* mutant or vice versa resulted in strongly impaired stem or petal growth due to reduced cell expansion. Given the known roles of AtKIN13A in ensuring an even distribution of Golgi stacks in the cells [27], [29] and in depolymerizing microtubules to determine the pattern of cell-wall deposition [31], [32], as well as our evidence for a function in modulating cell-wall synthesis, we conclude that the activity of the two *AtKIN13* genes is essential for normal cell-wall synthesis and thus plant-cell growth.

Why do different scenarios of reducing *AtKIN13* function lead to different outcomes, i.e. increased cell expansion in one case, reduced cell expansion in the other? We propose that when *AtKIN13* activity is only moderately reduced, as in the *atkin13a* mutants, the increased cell expansion represents a secondary effect due to triggering of the *THE1*-dependent signaling pathway by an initial cell wall-related defect caused by the *atkin13a* mutation; thus, in this case the secondary phenotype would outweigh the effect of the primary defect. Upon a stronger reduction in *AtKIN13* activity when both paralogues are compromised, the primary defect in cell-wall synthesis due to the *atkin13* mutation would be so severe in restricting cell growth as to outweigh the secondary effect of the *THE1*-dependent pathway, which is likely also triggered in these cases.

### *AtKIN13A* interacts with *AN* in controlling plant-cell growth

Given their opposite effects on trichome branching, *AN* and *AtKIN13A* had been proposed to act in a common pathway to target cell-wall loosening enzymes to sites of branch initiation [30]. A role for *AN* in regulating Golgi function as a plant orthologue to animal BARS has recently been demonstrated [35]. If the extra trichome branching seen in *atkin13a* mutants were indeed due to ectopic secretion from the clustered Golgi stacks, interfering with this secretion by blocking *AN* function would be expected to abrogate the increased branch formation. We find genetic evidence supporting this notion (Fig. 3). The *an* mutation is epistatic to the *atkin13a* mutation regarding trichome branching, suggesting that the clustering of Golgi stacks that results from loss of *AtKIN13A* function does not lead to the formation of extra branches in the absence of *AN* activity.

Regarding phenotypes other than trichome branching, the interpretation of the genetic interaction between *AN* and *AtKIN13A* is less straightforward (Fig. 3). Double mutant plants show a synergistic phenotype with respect to rosette growth and the organization of the inflorescence, while their cellular phenotype in petals is rather additive. Thus, different developmental or growth processes may depend differently on an even distribution of Golgi stacks in the cells versus efficient secretion from these Golgi stacks; alternatively, AtKIN13A may transport different cargo molecules or vesicles in different cells.

### *AtKIN13A* and the control of post-mitotic cell expansion

Reduced *AtKIN13* function in *atkin13a* mutants causes excess expansion of petal cells to a larger volume (Fig. 2). This increased cell expansion is accompanied by the deposition of more cell-wall material and dry matter, yet it does not coincide with ectopic endoreduplication. It is unclear at present, however, whether the increased cell-wall synthesis drives the excess cell expansion, or whether stronger cell expansion causes the increased cell-wall synthesis as a consequence. How can reduced activity of a factor that appears to be required for normal Golgi distribution and cell growth lead to enhanced cell expansion? The strongest evidence in this regard comes from our double mutant analysis with *the1* (Fig. 4,5). Loss of *THE1* activity abolishes not only the excess cell expansion of *atkin13a* mutant petal cells, but also the structural alterations of the cell wall as detected by FTIR analysis and the suppressed expression of primary cell wall-associated CesA subunit genes. Thus, reduced *AtKIN13A* activity appears to trigger signalling via the *THE1* pathway, but not the *WAK2* pathway, resulting in larger cells and an altered cell-wall structure. This reflects the two functions that have been previously ascribed to *THE1* in cell-wall integrity signalling and in promoting cell expansion [41], [42]. Surprisingly, however, the *THE1*-dependent altered cell-wall structure and reduced *CesA* gene expression in *atkin13a* mutants do not seem to be accompanied by obvious biochemical changes in cell-wall composition or in cellulose-microfibril arrangement, as our monosaccharide profiling and Scarlet 4B staining did not detect any significant differences.

At present it is unclear what triggers activation of the *THE1* signalling pathway in *atkin13a* mutants; yet, given the known role of *AtKIN13A* in dispersing the Golgi stacks within the cells [27], it is conceivable that an uneven Golgi distribution results in spatially uneven cell-wall deposition that sets off *THE1* signalling, potentially due to altered mechanical properties. The proposition that altered *AtKIN13A* activity modifies the pattern of cell-wall deposition has been demonstrated in the case of xylem cells [31], [32]. However, if also true for petal cells, this effect would appear to be rather subtle, as our cross-sections through the petal cells did not uncover gross unevenness in cell-wall thickness.

In conclusion, we have identified an essential role for *AtKIN13* activity in plant-cell growth. Altering this activity changes the amount of the cell wall that is deposited and causes enhanced or decreased cell expansion. In particular, a moderate reduction in *AtKIN13* activity leads to increased cell expansion via activation of the *THE1*-dependent cell-wall integrity pathway. Stronger impairment of *AtKIN13* function, however, causes reduced cell expansion, most likely due to impaired microtubule depolymerization and Golgi function. Thus, our results support an important role of the cell wall in limiting cell expansion and thus final cell and organ size in plants, and they provide a compelling case for the functional interplay between cell-wall integrity sensing and cell expansion.

## Methods

### Plant material and growth conditions

Arabidopsis Thaliana (L.) Heynh was used for this study. *atkin13a-2* (SAIL_761_B04), *atkin13a-4* (SALK_023418) and *atkin13b-1* (SAIL_60_C07), *the1-4* (SAIL_683_H03) and *wak2-1* (SAIL_286_E03) mutants were obtained from the Nottingham Arabidopsis Stock Centre (NASC; http://arabidopsis.info/). The *an-2* mutant has been described [34]. *atkin13a-3* was originally in the Landberg *erecta* background from the EMS screen, and was introgressed into the Col-0 background by back-crossing four times. Col-0 and L*er* were used as wild-type lines. T-DNA insertions and genotypes were confirmed by PCR amplification by using specific primers as described in the SIGnAL database (http://signal.salk.edu, Supplemental Table S1). The plants were grown under 16 h day∶8 h night conditions with fluorescent illumination (approximately 48 µmol m−2 sec−1) at 22°C.

### Genetic mapping

To map the *atkin13a-3* mutation, *atkin13a-3* in Landsberg *erecta* was crossed to Col-0 and the resulting F2 population was used for mapping using molecular markers available from The Arabidopsis Information Resource (TAIR; www.arabidopsis.org), mostly ones based on the Cereon polymorphism database (https://www.arabidopsis.org/browse/Cereon/index.jsp; [59]). For the fine-mapping, 526 F2 individuals were used; note that not all of these were genotyped for the more distant markers, explaining the different numbers of recombinants found in intervals I, II and III in Fig. S1. For critical recombinants, progeny testing was performed to verify the genotype at the mutant locus by analyzing the segregation of the phenotype in the progeny. A summary of the mapping is shown in Fig. S1, and the primers used are given in Supplemental Table S1.

### Phenotypic analysis

Organ and cell sizes were measured as described [44], [60]. Values are represented as mean + SD. Each value corresponds to at least ten petals from at least five plants. Hypocotyl elongation was measured after growing seedlings for 8 days in the dark on MS plates.

### Molecular cloning and genetic transformation

Constructs for plant transformation were generated, and plant transformation was performed by using standard techniques [61]. Oligonucleotides used are indicated in Supplemental Table S1. For mutant complementation, the *AtKIN13A* genomic sequence was amplified using primers pAtKIN13A_FOR_SacI and AtKIN13A_REV_PstI, subcloned into ML939 and transferred from there into a pBar-derivative [62]. Generation of artificial microRNA constructs was performed as described (http://wmd3.weigelworld.org/downloads/Cloning_of_artificial_microRNAs.pdf), and assembled amiRNAs were ligated to the *AlcA* promoter and inserted into a plant transformation vector derived from pBar containing *Pro35S:AlcR—ProAlcA:CFP* cassettes. The resulting construct thus allows simultaneous induction of *amiRNA* and *CFP* expression throughout the plant by EtOH treatment.

### Transmission and freeze-fracture scanning electron microscopy

Petals were fixed in 2.5% (v/v) glutaraldehyde and embedded in LR White resin (London Resin Company, Reading, Berkshire, UK) as described [63]. The material was sectioned with a diamond knife using a Leica UC6 ultramicrotome (Leica, Milton Keynes). Semi-thin sections of approx. 500 nm were stained with Toluidine blue for light microscopy, whereas ultrathin sections of approx. 90 nm were collected for electron microscopy. These were picked up on copper grids which had been pyroxylin and carbon-coated. The sections were stained with 2% (w/v) uranyl acetate and 2% lead citrate and viewed in a FEI Tecnai 20 transmission electron microscope (FEI UK Ltd, Cambridge, UK) at 200 kV. TIF digital image files were recorded using an AMT XR60 digital camera (Deben, Bury St Edmunds, UK).

Petals were cryo-fixed and freeze-fractured as described [64], using an ALTO 2500 cryo-system (Gatan, Oxford, England) attached to a Zeiss Supra 55 VP FEG scanning electron microscope (Zeiss SMT, Germany). The sample was imaged at 3 kV and digital TIF files were stored.

### Dry-weight analysis

To prepare cell wall materials, petals were freeze-dried and milled by rapid shaking with a ball bearing for 20 min and washed by 70% of ethanol, then rinsed by methanol-chloroform (1∶1, v/v). Cell wall materials were dried by using vacuum dryer for 2d and measured.

### Fourier-transform infrared spectroscopy

More than ten 20-day old leaves were pre-cleaned by treating with 70% ethanol, followed by methanol:chloroform (1∶1, v:v) and finally acetone, air-dried, and homogenized by ball milling. The dry cell-wall material was loaded between two CaF_2_ windows. The spectra were obtained with a Perkin Elmer GX 2000 FTIR spectrometer [65]. For each sample 128 scans were co-added to increase the signal-to-noise ratio. Spectra were background corrected by subtraction of the blank. Using the Spectrum 5.0.1 software the spectra were baseline-corrected and normalized. Spectra were analyzed by the covariance-matrix approach for Principal Component analysis [66]. Exploratory Principal Component Analysis has been used before to characterize differences in FTIR spectra [67]; this approach derives variables, so-called Principal Components (PCs), that quantify the variance in data sets of high dimensionality, such as FTIR spectra.

### Cell wall biochemical analyses

The cell wall biochemical assay was done as described [68]. Briefly, cell wall monosaccharides were assayed after hydrolysis with 2M trifluoroacetic acid (TFA) as alditol acetate by gas chromatography performed on an Agilent 6890N GC System coupled with an Agilent 5973N Mass Selective Detector (Waldbronn, Germany). *Myo*-Inositol was added as an internal standard. Cellulose was determined on the fraction resistant to extraction with 2M TFA using glucose equivalents as standard by anthrone assay [69]. Uronic acids were colorimetrically quantified using the soluble 2M TFA fraction using 2-hydroxydiphenyl as reagent and galacturonic-acid as standard [70]. Statistical significance was determined using Student's t-test

### RNA isolation and qRT-PCR analysis

For qRT-PCR analysis, total RNA was extracted using a TRIzol (Invitrogen) from inflorescences of more than five plants per sample. Extracted RNA was treated with TURBO DNA-free kit (Invitrogen). First-strand cDNA was prepared using the SuperScript first-strand synthesis system for RT-PCR (Invitrogen). Quantitative RT-PCR analysis was performed on a LightCycler LC480 (Roche).

### Accession numbers

AGI codes of genes discussed in this paper are: *AtKIN13A* (At3g16630), *AtKIN13B* (At3g16060), *THE1* (At5g54380), *WAK2* (At1g21270), *AN* (At1g01510), *CesA1* (At4g32410), *CesA3* (At5g05170), *CesA6* (At5g64740).

## Supporting Information

Figure S1 Genetic mapping of the *atkin13a-3* mutation. Summary of mapping the large-petal mutation on chromosome 3. Names of molecular markers used and their positions on the genetic and physical maps are indicated, as well as the number of recombinants found for each marker (bold red). For the fine-mapping to define the location of the mutated gene in interval III, 526 individuals were tested; note that not all of these were genotyped for the more distant markers, explaining the different numbers of recombinants found in intervals I, II and III. The final mapping interval was less than 40 kb. CER markers are from the Monsanto Cereon collection (https://www.arabidopsis.org/browse/Cereon/index.jsp; [59]). See Methods for further information.
(TIF)

Figure S2 Cell size phenotypes and molecular nature of *atkin13a* mutants. (A) Expression of full-length *AtKIN13A* and *AtKIN13B* in the indicated genotypes as determined by RT-PCR. (B) Gel print of a wild-type petal and magnification of cells from the indicated regions of the petal. (C) Average cell length of cells in the apical and middle regions of two petals from the indicated genotypes. Individual cell-length values from the cells marked by a black line in (D) were averaged. Standard deviation is shown. (D) Measurements of individual cell lengths along the longitudinal axis of two petals per genotype. (E) Petal size and petal-cell size of the indicated genotypes. (F) Frequency distributions of trichomes with the indicated numbers of branches from the different genotypes shown. n>100 trichomes per genotype. (G) Light micrographs of representative trichomes from *atkin13a-3* (top) and *atkin13a-4* mutant leaves (bottom). (H) Schematic representation of the *AtKIN13A* cDNA. Black bars represent 5′ and 3′ UTRs and red arrow shows coding sequence. The region encoding the motor domain is indicated by the black bar below. Also shown is a partial sequence alignment of KIN13 proteins from various organisms. The invariant DLL sequence, of which the first leucine is mutated to a phenylalanine in *atkin13a-3* is highlighted. Asterisk indicates significant difference from wild-type at p<0.05 (with Bonferroni correction).
(TIF)

Figure S3 Complementation of the *atkin13a-3* mutation by a genomic *AtKIN13A* transgene. (A) Petal size of the indicated genotypes relative to wild type. Values are mean + SD from 16 petals per genotype. (B) Petal-cell size of the indicated genotypes. Values are mean + SD from 500 petal cells from 10 petals per genotype. (C) Representative trichome from a complemented line in the *atkin13a-3* background. Asterisk indicates significant difference at p<0.05.
(TIF)

Figure S4 Cell-expansion and ploidy phenotypes of *atkin13a* mutants. (A) Low-magnification transmission-electron micrograph of a wild-type petal (left) and high-magnification transmission-electron micrographs of wild-type and *atkin13a-3* petals (right), showing the basal walls of conical cells on the adaxial petal surface. Lengths of scale bars are indicated. (B) Thickness of the basal walls of conical cells as determined from transmission-electron micrographs. Values are mean ± SD from 200 petal cells from 10 petals. (C) Optical transverse section through an mPS-PI stained wild-type petal imaged by confocal microscopy (left) and average heights of conical petal cells in the indicated genotypes. Values are mean ± SD from 200 petal cells from 10 petals. (D) Leaf phenotypes of *atkin13a* and *atkin13b* mutants. Micrographs show leaf mesophyll cells, with cell outlines highlighted in white. Also shown are the outlines of mature leaves, and measurements of leaf-cell sizes and numbers. Values are mean + SD from 200 leaf cells from 10 leaves. Cell numbers were calculated by dividing average leaf area by the average leaf-cell area. (E) Ploidy measurements of nuclei from petal cells indicate no difference between wild-type and *atkin13a-3* mutants.
(TIF)

Figure S5 Genetic interactions of *AtKIN13A.* (A–C) Petal sizes of the indicated genotypes. (A) *atkin13a-3 ein2* double mutant. (B) *atkin13a-3 rot3-1* double mutant. (C) *atkin13a-3 bri1-5* double mutant. Values are mean ± SD of 500 petals from 10 plants.
(TIF)

Figure S6 Exploratory Principal Component Analysis separates the different genotypes and their biological replicates. (A)A plot of the first two principal components (PCs) shows that the samples can be separated by PC1 into groups (red dashed line); (a) Col-0, *the1-4*, and *atkin13a-3 the1-4* and (b) *atkin13a-3* and *atkin13a-4*. (B) Loading of PC1 along the wavenumbers in the full infrared data sets obtained for the five genotypes. (C) Frequency of trichomes with the indicated number of branches in the four genotypes shown.
(TIF)

Figure S7 Scarlet 4B staining does not reveal a difference in cellulose-microfibril organization in *atkin13a* mutants versus wild type. Fluorescence micrographs of Scarlet 4B-stained wild-type (left) and *atkin13a-3* (right) petal cells taken from the top, middle and bottom regions of the petal.
(TIF)

Figure S8 Effect of downregulating *AtKIN13A* expression in the *atkin13b-1* mutant background. (A) CFP fluorescence micrograph showing successful induction of amiRNA expression after EtOH-induction. (B) Petal size is reduced upon downregulation of *AtKIN13A* expression in the *atkin13b-1* mutant background by EtOH-induction. Values are mean ± SD of 500 petals from 10 plants. Asterisk indicates significant difference from wild-type at p<0.05 (with Bonferroni correction).
(TIF)

Table S1 List of oligonucleotides used. Sequences and usage of oligonucleotides employed in this study are given.
(DOCX)
