# Supplementary figures and images for: Atkinesin-13A Modulates Cell-Wall Synthesis and Cell Expansion in Arabidopsis thaliana via the THESEUS1 Pathway
Source: PLoS Genet. 2014 Sep 18;10(9):e1004627. doi: 10.1371/journal.pgen.1004627 (PMC4169273; doi:10.1371/journal.pgen.1004627)

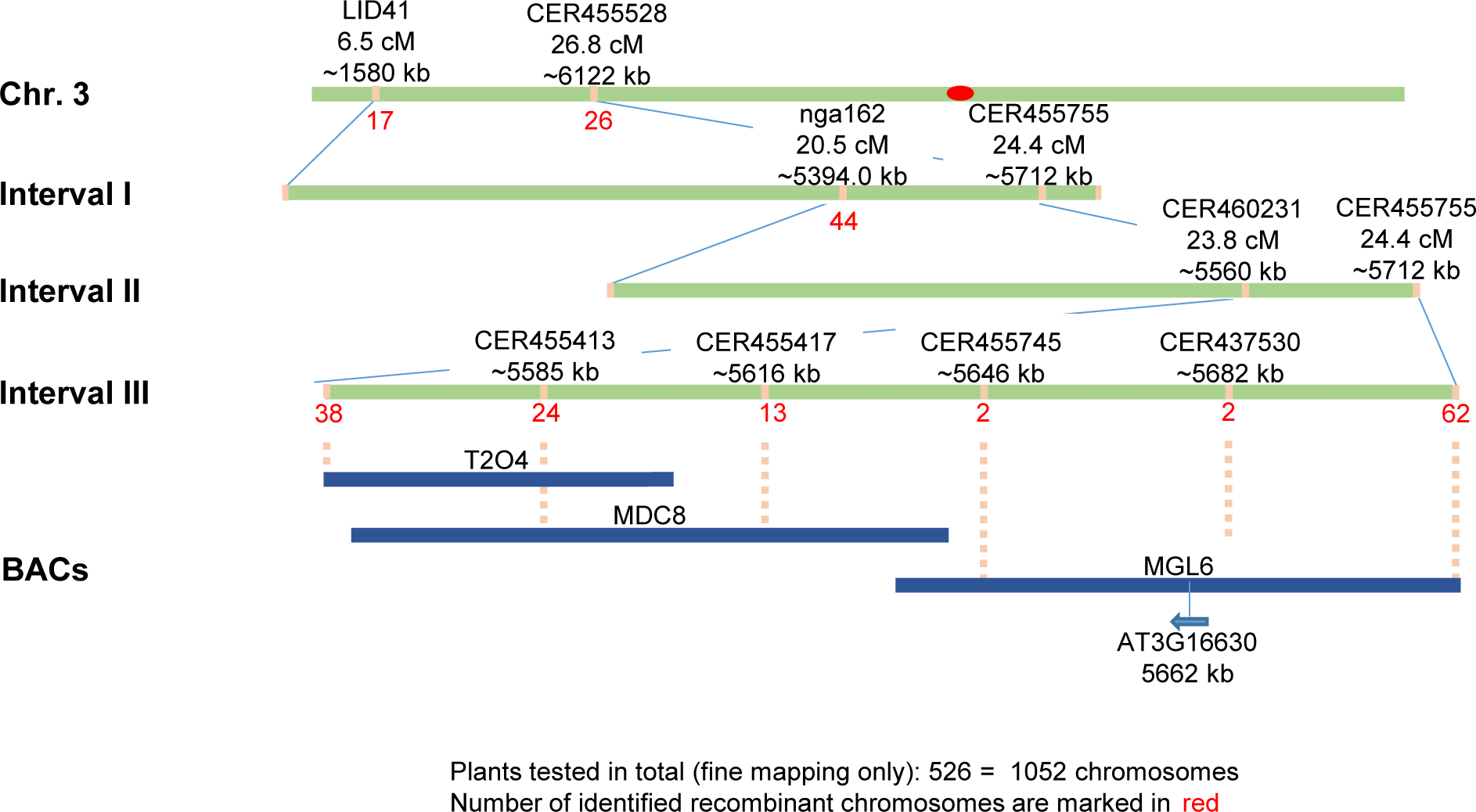

Supplement: Figure S1 — Genetic mapping of the atkin13a-3 mutation. Summary of mapping the large-petal mutation on chromosome 3. Names of molecular markers used and their positions on the genetic and physical maps are indicated, as well as the number of recombinants found for each marker (bold red). For the fine-mapping to define the location of the mutated gene in interval III, 526 individuals were tested; note that not all of these were genotyped for the more distant markers, explaining the different numbers of recombinants found in intervals I, II and III. The final mapping interval was less than 40 kb. CER markers are from the Monsanto Cereon collection (https://www.arabidopsis.org/browse/Cereon/index.jsp; [59]). See Methods for further information. (TIF) [file pgen.1004627.s001.tif]

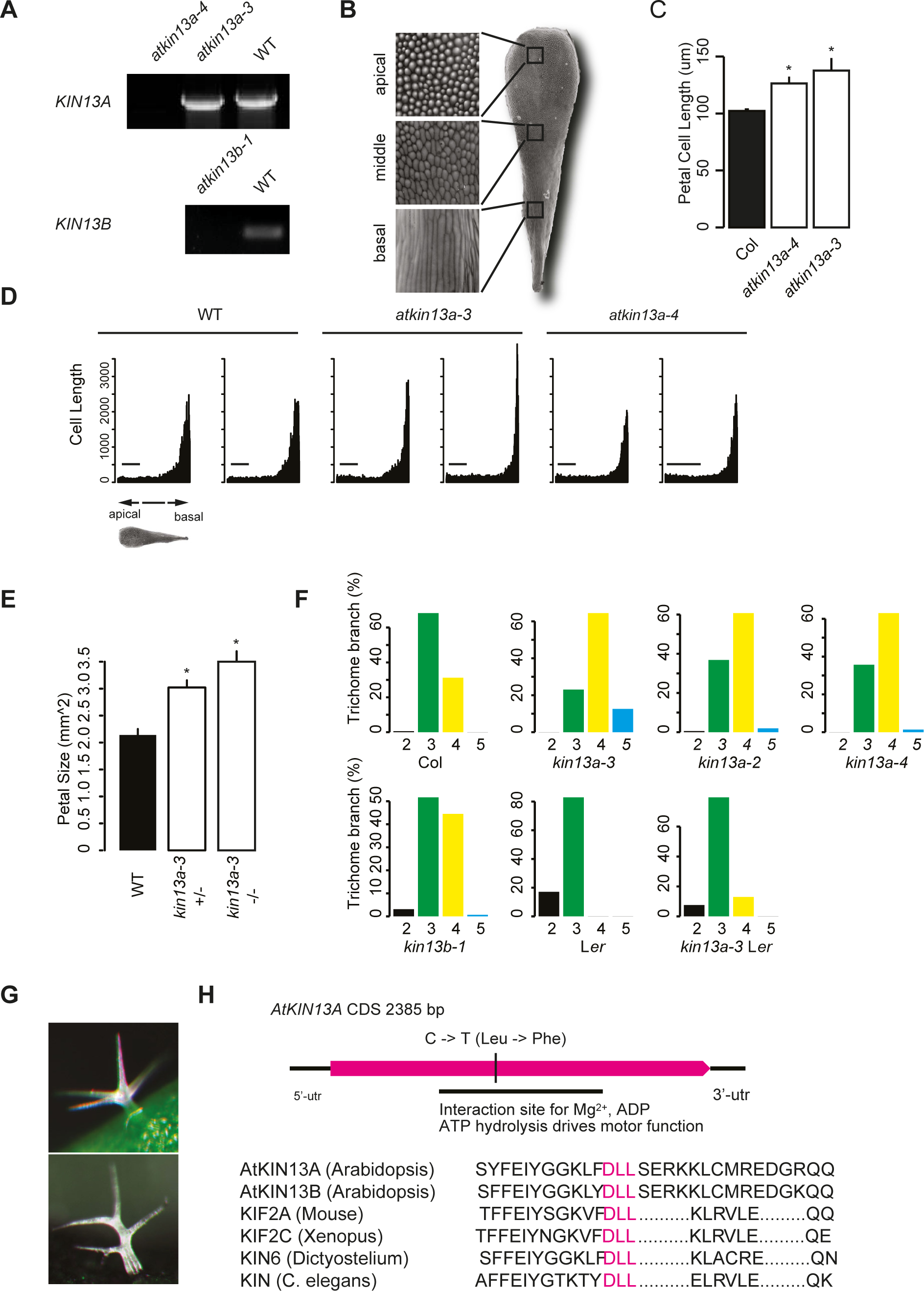

Supplement: Figure S2 — Cell size phenotypes and molecular nature of atkin13a mutants. (A) Expression of full-length AtKIN13A and AtKIN13B in the indicated genotypes as determined by RT-PCR. (B) Gel print of a wild-type petal and magnification of cells from the indicated regions of the petal. (C) Average cell length of cells in the apical and middle regions of two petals from the indicated genotypes. Individual cell-length values from the cells marked by a black line in (D) were averaged. Standard deviation is shown. (D) Measurements of individual cell lengths along the longitudinal axis of two petals per genotype. (E) Petal size and petal-cell size of the indicated genotypes. (F) Frequency distributions of trichomes with the indicated numbers of branches from the different genotypes shown. n>100 trichomes per genotype. (G) Light micrographs of representative trichomes from atkin13a-3 (top) and atkin13a-4 mutant leaves (bottom). (H) Schematic representation of the AtKIN13A cDNA. Black bars represent 5′ and 3′ UTRs and red arrow shows coding sequence. The region encoding the motor domain is indicated by the black bar below. Also shown is a partial sequence alignment of KIN13 proteins from various organisms. The invariant DLL sequence, of which the first leucine is mutated to a phenylalanine in atkin13a-3 is highlighted. Asterisk indicates significant difference from wild-type at p<0.05 (with Bonferroni correction). (TIF) [file pgen.1004627.s002.tif]

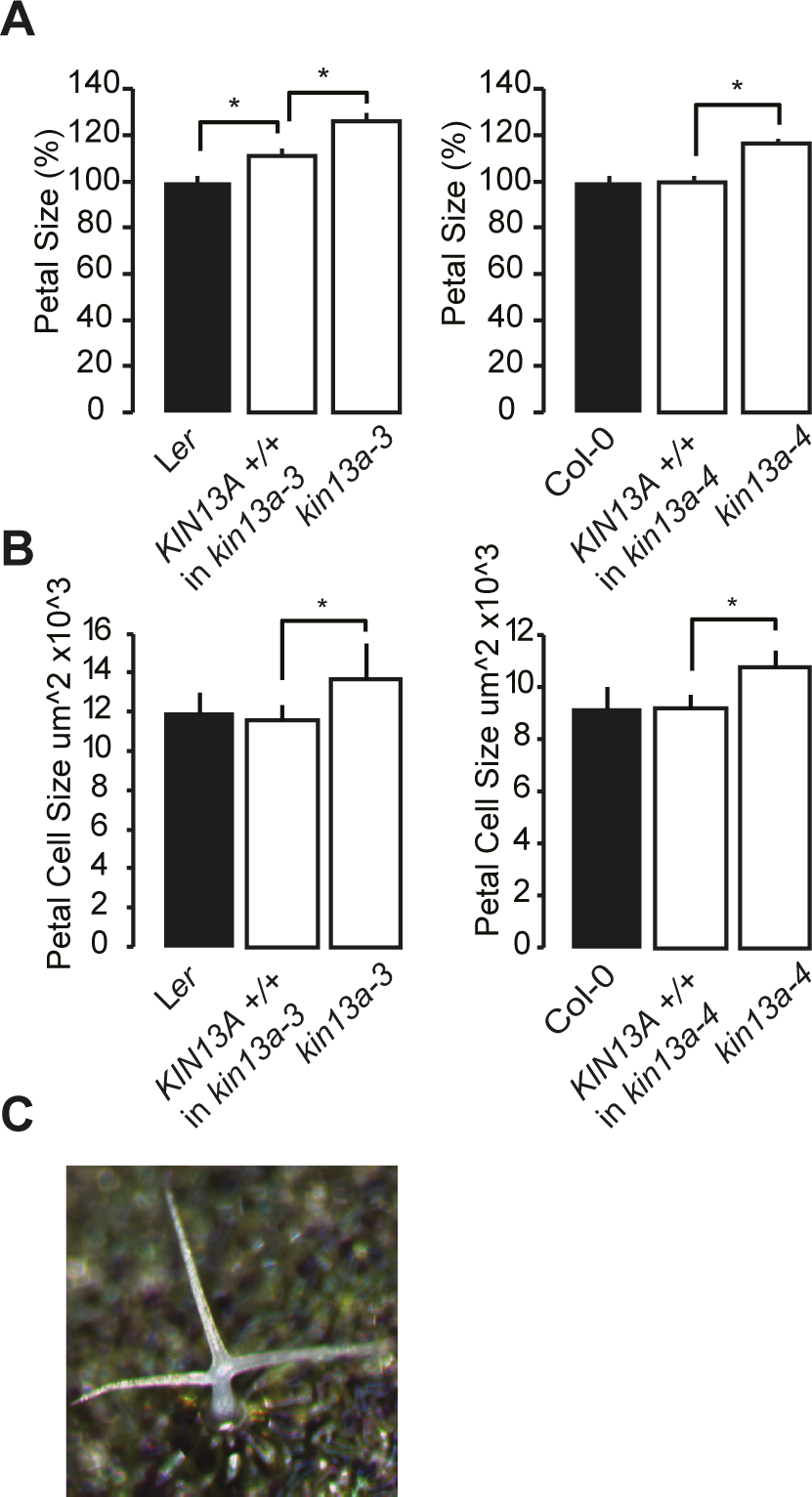

Supplement: Figure S3 — Complementation of the atkin13a-3 mutation by a genomic AtKIN13A transgene. (A) Petal size of the indicated genotypes relative to wild type. Values are mean + SD from 16 petals per genotype. (B) Petal-cell size of the indicated genotypes. Values are mean + SD from 500 petal cells from 10 petals per genotype. (C) Representative trichome from a complemented line in the atkin13a-3 background. Asterisk indicates significant difference at p<0.05. (TIF) [file pgen.1004627.s003.tif]

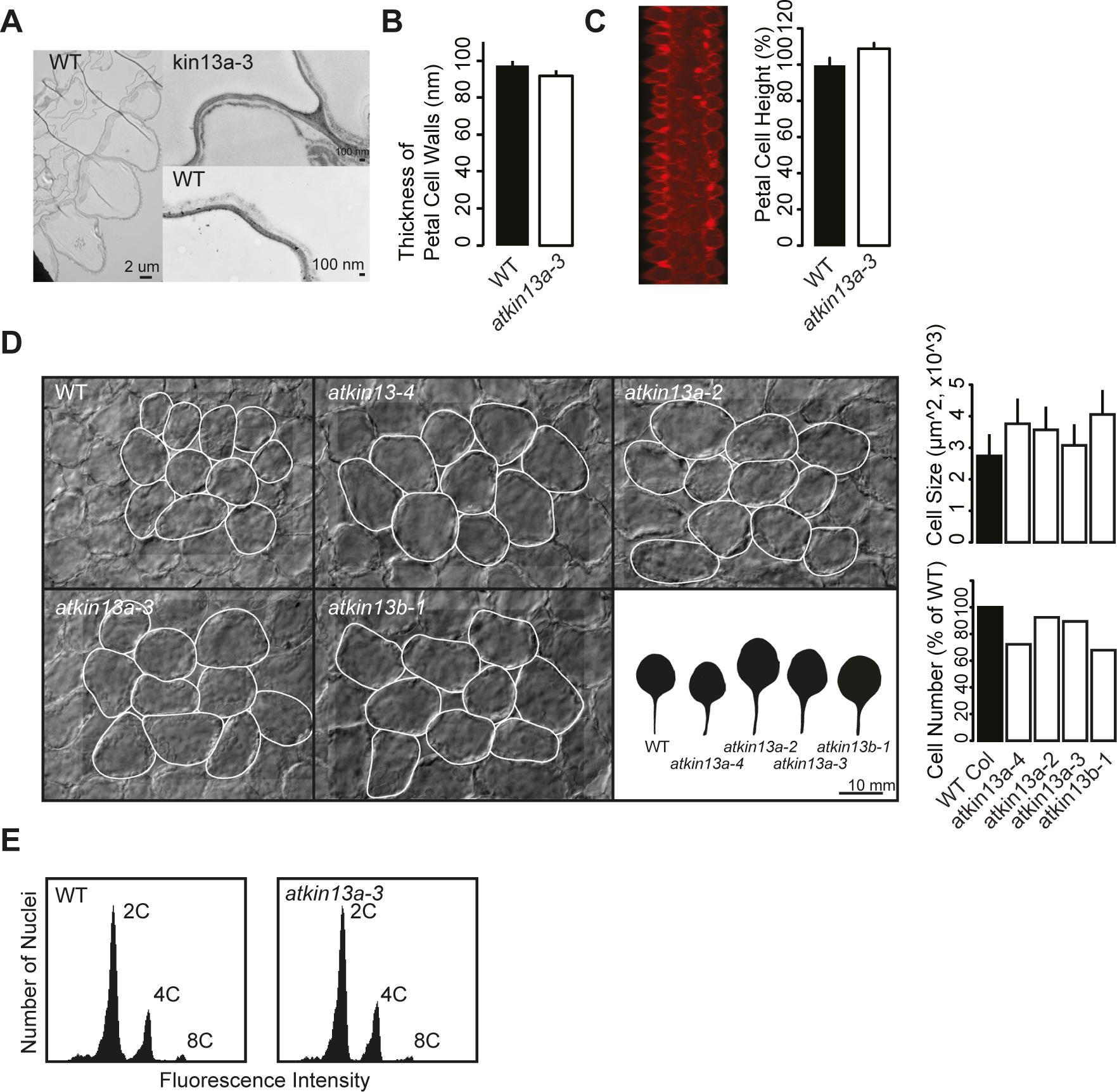

Supplement: Figure S4 — Cell-expansion and ploidy phenotypes of atkin13a mutants. (A) Low-magnification transmission-electron micrograph of a wild-type petal (left) and high-magnification transmission-electron micrographs of wild-type and atkin13a-3 petals (right), showing the basal walls of conical cells on the adaxial petal surface. Lengths of scale bars are indicated. (B) Thickness of the basal walls of conical cells as determined from transmission-electron micrographs. Values are mean ± SD from 200 petal cells from 10 petals. (C) Optical transverse section through an mPS-PI stained wild-type petal imaged by confocal microscopy (left) and average heights of conical petal cells in the indicated genotypes. Values are mean ± SD from 200 petal cells from 10 petals. (D) Leaf phenotypes of atkin13a and atkin13b mutants. Micrographs show leaf mesophyll cells, with cell outlines highlighted in white. Also shown are the outlines of mature leaves, and measurements of leaf-cell sizes and numbers. Values are mean + SD from 200 leaf cells from 10 leaves. Cell numbers were calculated by dividing average leaf area by the average leaf-cell area. (E) Ploidy measurements of nuclei from petal cells indicate no difference between wild-type and atkin13a-3 mutants. (TIF) [file pgen.1004627.s004.tif]

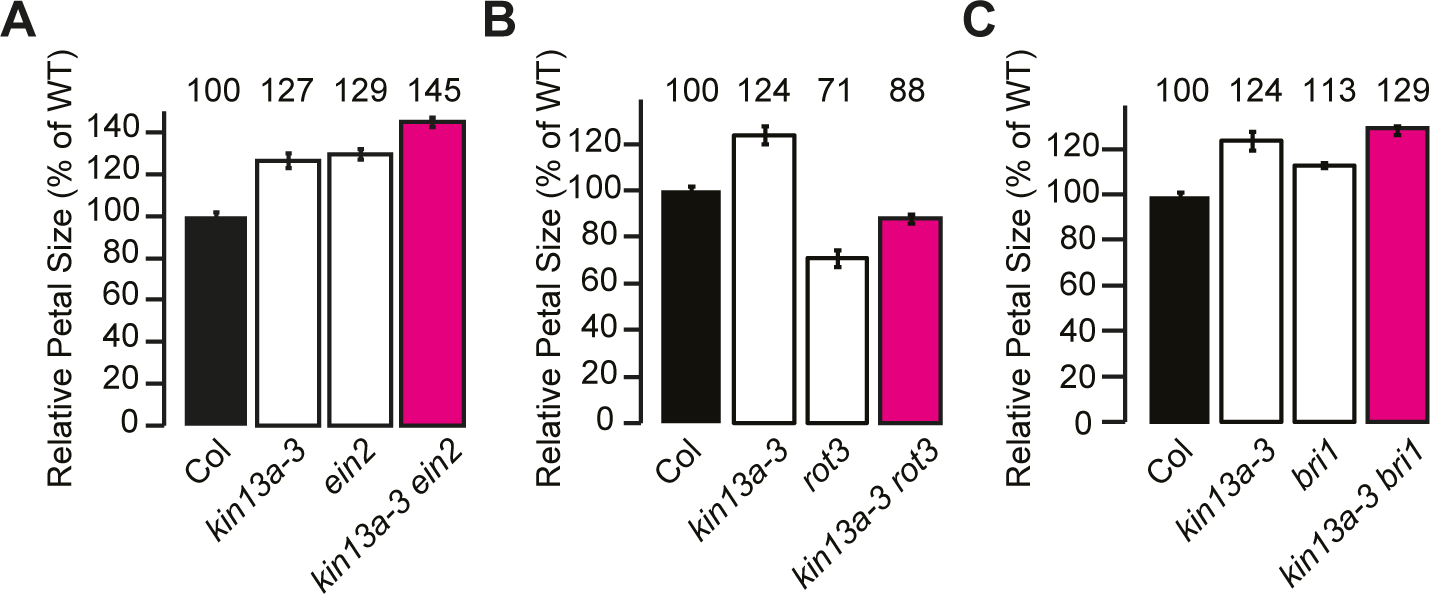

Supplement: Figure S5 — Genetic interactions of AtKIN13A. (A–C) Petal sizes of the indicated genotypes. (A) atkin13a-3 ein2 double mutant. (B) atkin13a-3 rot3-1 double mutant. (C) atkin13a-3 bri1-5 double mutant. Values are mean ± SD of 500 petals from 10 plants. (TIF) [file pgen.1004627.s005.tif]

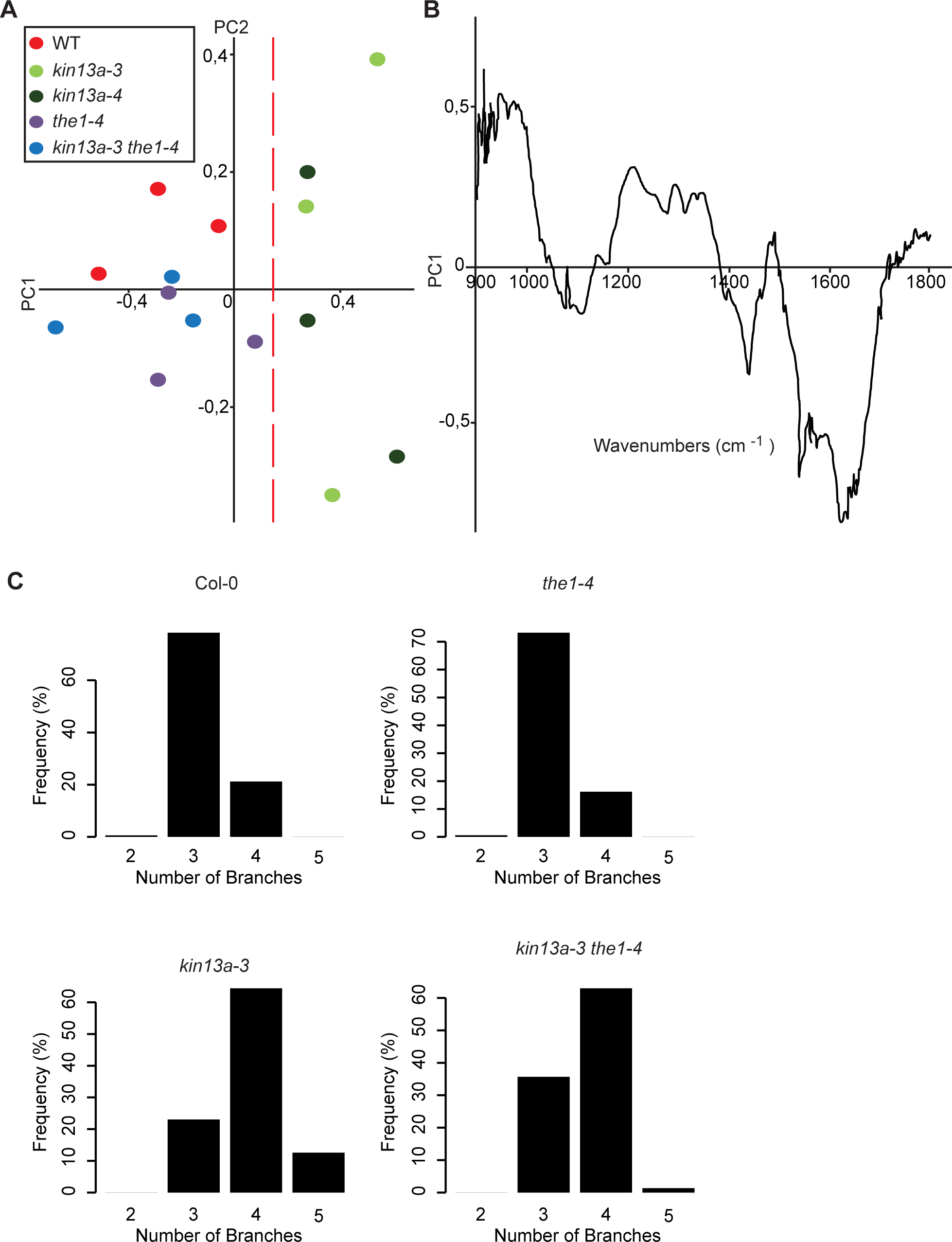

Supplement: Figure S6 — Exploratory Principal Component Analysis separates the different genotypes and their biological replicates. (A)A plot of the first two principal components (PCs) shows that the samples can be separated by PC1 into groups (red dashed line); (a) Col-0, the1-4, and atkin13a-3 the1-4 and (b) atkin13a-3 and atkin13a-4. (B) Loading of PC1 along the wavenumbers in the full infrared data sets obtained for the five genotypes. (C) Frequency of trichomes with the indicated number of branches in the four genotypes shown. (TIF) [file pgen.1004627.s006.tif]

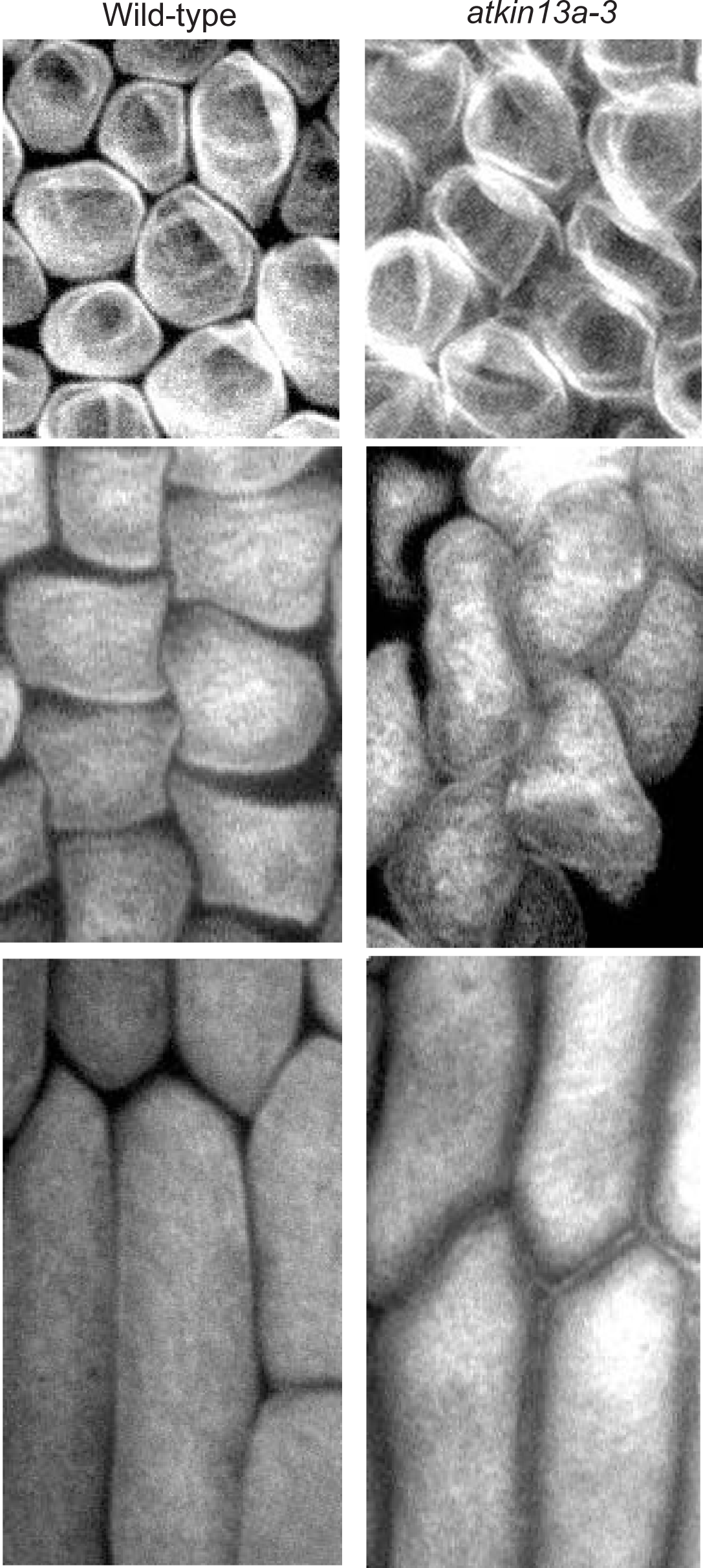

Supplement: Figure S7 — Scarlet 4B staining does not reveal a difference in cellulose-microfibril organization in atkin13a mutants versus wild type. Fluorescence micrographs of Scarlet 4B-stained wild-type (left) and atkin13a-3 (right) petal cells taken from the top, middle and bottom regions of the petal. (TIF) [file pgen.1004627.s007.tif]

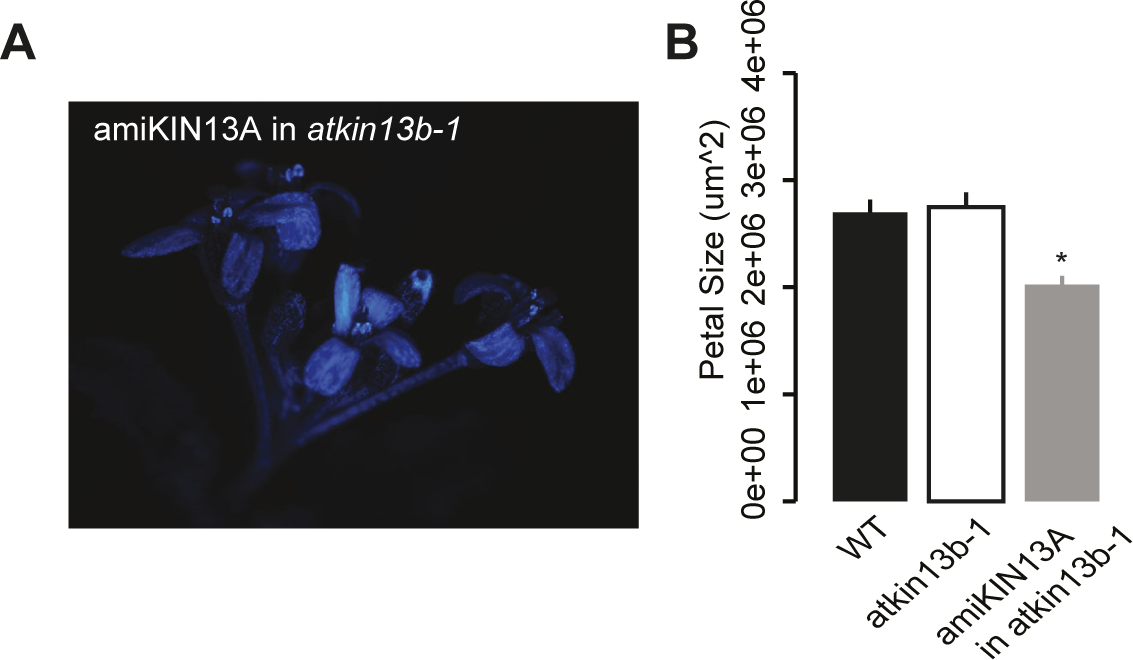

Supplement: Figure S8 — Effect of downregulating AtKIN13A expression in the atkin13b-1 mutant background. (A) CFP fluorescence micrograph showing successful induction of amiRNA expression after EtOH-induction. (B) Petal size is reduced upon downregulation of AtKIN13A expression in the atkin13b-1 mutant background by EtOH-induction. Values are mean ± SD of 500 petals from 10 plants. Asterisk indicates significant difference from wild-type at p<0.05 (with Bonferroni correction). (TIF) [file pgen.1004627.s008.tif]
