# Supplementary material for: Atkinesin-13A Modulates Cell-Wall Synthesis and Cell Expansion in Arabidopsis thaliana via the THESEUS1 Pathway
Source: PLoS Genet. 2014 Sep 18;10(9):e1004627. doi: 10.1371/journal.pgen.1004627 (PMC4169273; doi:10.1371/journal.pgen.1004627)
Supplement: Table S1 — List of oligonucleotides used. Sequences and usage of oligonucleotides employed in this study are given. (DOCX) [file pgen.1004627.s009.docx]

**Supplemental Table 1: List of oligos used**

| **Oligonucleotide name** | **Usage** | **Sequence (5’ – 3’)** |
| --- | --- | --- |
| pAtKIN13A_FOR_SacI | for complementation test | TTGAGCTCGTCCTGTGTTTCGTCGTCGTC |
| AtKIN13A_REV_PstI |  | TCTGCAGAGATCACCCAACCAACCTTTCACAC |
| AtKIN13A_FOR_HindIII_BamH | for CDS cloning | AAGAAGCTTGGATCCTCTCATGGGCGGCCAAATGCA |
| amiRNA KIN13A miR-s | amiKIN13A | GATTCTCGACCACCCTCCTCCTGTCTCTCTTTTGTATTCC |
| amiRNA KIN13A miR-a |  | GACAGGAGGAGGGTGGTCGAGAATCAAAGAGAATCAATGA |
| amiRNA KIN13A miR-*s |  | GACAAGAGGAGGGTGCTCGAGATTCACAGGTCGTGATATG |
| amiRNA KIN13A miR-*a |  | GAATCTCGAGCACCCTCCTCTTGTCTACATATATATTCCT |
| qRT_KIN13B_F | for qRT-PCR of KIN13B | ACCGTCTTGCTCATTTCCAG |
| qRT_KIN13B_R |  | ACAAGTGCAAAACCCGAAAC |
| qRT_CesA1_F | for qRT-PCR of CesA1 | AGAAGATGTGGACCCAAATGA |
| qRT_CesA1_R |  | GGACAACAAGTTCCAACATTACA |
| qRT_CesA3_F | for qRT-PCR of CesA3 | CCCTTCACTAGCCGAGTCAC |
| qRT_CesA3_R |  | TTTTTAACCCTCAAAACAGGTAAA |
| qRT_CesA6_F | for qRT-PCR of CesA6 | GACCTCTCTACCGCTCATCG |
| qRT_CesA6_R |  | ATCATCGATCCCAACTTTGC |
| KIN13A_qRT_F | for qRT-PCR of KIN13A | CCGGGAAGCATGATAGAAAA |
| KIN13A_qRT_R |  | CCCAACCAACCTTTCACACT |
| SAIL_286_E03_LP | for wak2-1 genotyping | CATGTGCTGTTACCACCACAC |
| SAIL_286_E03_RP |  | ATTCCTTGCAAGTTGCAACTG |
| SAIL_683_H03_LP | for the1-4 genotyping | CCGGGTCTAGATAACCAAAGC |
| SAIL_683_H03_RP |  | TGTTTTAACCGTTAGCGTTGG |
| kin13a-3_F | for kin13a-3 genotyping, MboI digest | GATTCCTCATTTGCTCCAGTTG |
| kin13a-3_R |  | TGCTAACAGATTCCAGTTTTTAGAGG |
| SAIL_60_C07_LP | for SAIL_60_C07_LP (KIN13B) genotyping | GCAGATCTAAGAGCCCAGGA |
| SAIL_60_C07_RP |  | CGAGTTTAAGGGAGCGTCAG |
| SAIL_761_B04_LP | for SAIL_761_B04 (kin13a) genotyping | AAAAAGGCATGTCACGTGATC |
| SAIL_761_B04_RP |  | CAAGTGAAACTGCTTCAAGGC |
| HBo036 (CER437530for) | map-based cloning | CAATAGCCCAGACTCTTTAG |
| HBo037 (CER437530rev) | map-based cloning | GCAACCTTCGATTTTCG |
| HBo038 (CER455745for) | map-based cloning | CAAACATACATAACCGATTC |
| HBo039 (CER455745rev) | map-based cloning | CCGAGTAAACTATGAAATTC |
| HBo040 (CER455417for) | map-based cloning | TAAATAGTCAACTGTTTGTTTG |
| HBo041 (CER455417rev) | map-based cloning | GGACCAACTACAATTGTTTC |
| HBo042 (CER455413for) | map-based cloning | TGATACTCTACTCGGATTTG |
| HBo043 (CER455413rev) | map-based cloning | GTGCCTTCAACTGCAGTA |
| nga162_for | map-based cloning | CATGCAATTTGCATCTGAGG |
| nga162_rev | map-based cloning | CTCTGTCACTCTTTTCCTCTGG |
| CER455755_for | map-based cloning | GTAATGTGTAGAATGTGACCAA |
| CER455755_rev | map-based cloning | TCCACCTAACCTAATAATAACAA |
| CER460231_for | map-based cloning | TTCACGCACATTCTATTTC |
| CER460231_rev | map-based cloning | TATATTTTATTTTCCTGCTCAA |
| LEo047 (CER455528_for) | map-based cloning | CAAGTTGATAGAGGGTGGTGCAGC |
| LEo048 (CER455528_rev) | map-based cloning | CCTCGCCCTACTCGCAACATAG |
